# Su(Hw) primes 66D and 7F *Drosophila* chorion genes loci for amplification through chromatin decondensation

**DOI:** 10.1038/s41598-021-96488-0

**Published:** 2021-08-20

**Authors:** Nadezhda E. Vorobyeva, Maksim Erokhin, Darya Chetverina, Alexey N. Krasnov, Marina Yu. Mazina

**Affiliations:** Institute of Gene Biology, Russian Academy of Sciences, Moscow, Russia 119334

**Keywords:** Origin selection, Chromatin, Chromatin structure

## Abstract

Suppressor of Hairy wing [Su(Hw)] is an insulator protein that participates in regulating chromatin architecture and gene repression in *Drosophila*. In previous studies we have shown that Su(Hw) is also required for pre-replication complex (pre-RC) recruitment on Su(Hw)-bound sites (SBSs) in *Drosophila* S2 cells and pupa. Here, we describe the effect of Su(Hw) on developmentally regulated amplification of 66D and 7F *Drosophila* amplicons in follicle cells (DAFCs), widely used as models in replication studies. We show Su(Hw) binding co-localizes with all known DAFCs in *Drosophila* ovaries, whereas disruption of Su(Hw) binding to 66D and 7F DAFCs causes a two-fold decrease in the amplification of these loci. The complete loss of Su(Hw) binding to chromatin impairs pre-RC recruitment to all amplification regulatory regions of 66D and 7F loci at early oogenesis (prior to DAFCs amplification). These changes coincide with a considerable Su(Hw)-dependent condensation of chromatin at 66D and 7F loci. Although we observed the Brm, ISWI, Mi-2, and CHD1 chromatin remodelers at SBSs genome wide, their remodeler activity does not appear to be responsible for chromatin decondensation at the 66D and 7F amplification regulatory regions. We have discovered that, in addition to the CBP/Nejire and Chameau histone acetyltransferases, the Gcn5 acetyltransferase binds to 66D and 7F DAFCs at SBSs and this binding is dependent on Su(Hw). We propose that the main function of Su(Hw) in developmental amplification of 66D and 7F DAFCs is to establish a chromatin structure that is permissive to pre-RC recruitment.

## Introduction

Suppressor of Hairy wing [Su(Hw)] was initially described as a DNA-binding protein responsible for the function of the *Drosophila* gypsy insulator^1^, which is capable of both enhancer blocking^2,3^ and barrier activity^4^, i.e. protection of the transgene from the effect of outer regulatory elements and the spread of repressive chromatin. The advent of high-throughput genome-wide analysis methods revealed that although some tested Su(Hw)-bound sites (SBSs) exhibited chromatin barrier activity, other SBSs failed to block enhancer-promoter interaction in transgenic assays^5,6^. In addition, more than half of SBSs that are depleted of CP190 and Mod(mdg4)67.2 cofactors repress transcription being placed near the promoter of a transgene^6^. The transcriptional repressor function of Su(Hw) was found to be responsible for the strongest phenotype of *su(Hw)* mutants—female sterility^7^. Su(Hw) binds to the promoter regions of some CNS-enriched target genes in *Drosophila* ovary to repress their transcription^7,8^. A decrease in the transcription of one of these genes, *rbp9,* successfully restored the fertility of *su(Hw)*^–/–^ mutants^7^.

We previously demonstrated that Su(Hw) is both necessary and sufficient for pre-replication complex (pre-RC) recruitment to SBSs in *Drosophila* S2 cells and pupa^9,10^. Prior to this the only known role of Su(Hw) in replication regulation was the demarcation of replication domains^11^. We proposed that Su(Hw) can act as a key factor influencing pre-RC formation at the SBSs by affecting chromatin structure. In accordance with our data, recent whole-genome studies of Su(Hw) binding in the *Drosophila* ovary revealed that a significant number of Su(Hw) sites are not involved in either repressive or insulator functions of the protein, and the authors suggested that these sites may be ascribed to a Su(Hw) replication function^12^.

In a preliminary study we identified SBSs in all currently annotated *Drosophila* amplicons in follicle cells (DAFCs) and described the contribution of Su(Hw) in recruiting the ORC3 subunit of the origin recognition complex (ORC) to SBSs in some of these amplicons^13^. DAFCs present one of the most well-established systems for investigating the regulation of metazoan replication origins in vivo^14–16^. Follicle cells are somatic cells that surround the oocyte and nurse cells during *Drosophila* oogenesis and secrete the chorion proteins that form the embryo shell. DAFCs contain genes encoding chorion proteins and other products necessary for normal egg shell formation^17,18^. At a certain stage of ovary development (in the egg chamber stages 10–13) these genome regions undergo repeated rounds of replication initiation to produce a gradient of increased DNA copy number. The same replication initiation proteins involved in genome replication during S-phase also participate in DAFC amplification^15^. Recent whole-genome analyses have revealed all DAFCs in the *Drosophila* genome and unveiled hidden aspects of their function^19^. Although the developmentally regulated gene amplification is mainly hypothesized as an avenue to achieve a high level of necessary gene products, there is no direct correlation between the rate of locus amplification and the transcription level of the genes within^19,20^. These data challenge the seemingly well-described model of DAFCs amplification and suggest the existence of some mechanism responsible for the regulation of the replication but not transcription of DAFCs.

Here, we expand our previous investigations of Su(Hw) function in replication. Although there is evidence that Su(Hw) plays a role in replication origin positioning, the direct impact of Su(Hw) on the replication process has so far not been demonstrated. To fill this gap, we describe a role for Su(Hw) in amplification and pre-RCs positioning in the well-characterized 66D and 7F *Drosophila* amplification loci.

## Results

### Su(Hw) is present at 66D and 7F chorion gene loci and affects their amplification

It is well known that female sterility is the main consequence of loss of Su(Hw) function during fly development^21,22^. The complete loss of Su(Hw) binding to chromatin (in *su(Hw)*^*v*/*2*^*, su(Hw)*^*v*/*E8*^*, su(Hw)*^*2*/*E8*^*, su(Hw)*^*Pb*/*v*^*, su(Hw)*^*Pb*/*2*^ heteroallelic mutant combinations) causes mid-stage arrest of oogenesis and egg chamber degeneration at approximately stage 9, while mutants with partially retained Su(Hw) binding (*su(Hw)*^*v*/*f*^) are fertile and demonstrate only defects in nurse cell chromosome structure. Thus we used the fertile *su(Hw)*^*v*/*f*^ mutants to evaluate the effect of Su(Hw) on DAFCs amplification.

**Figure 1:**
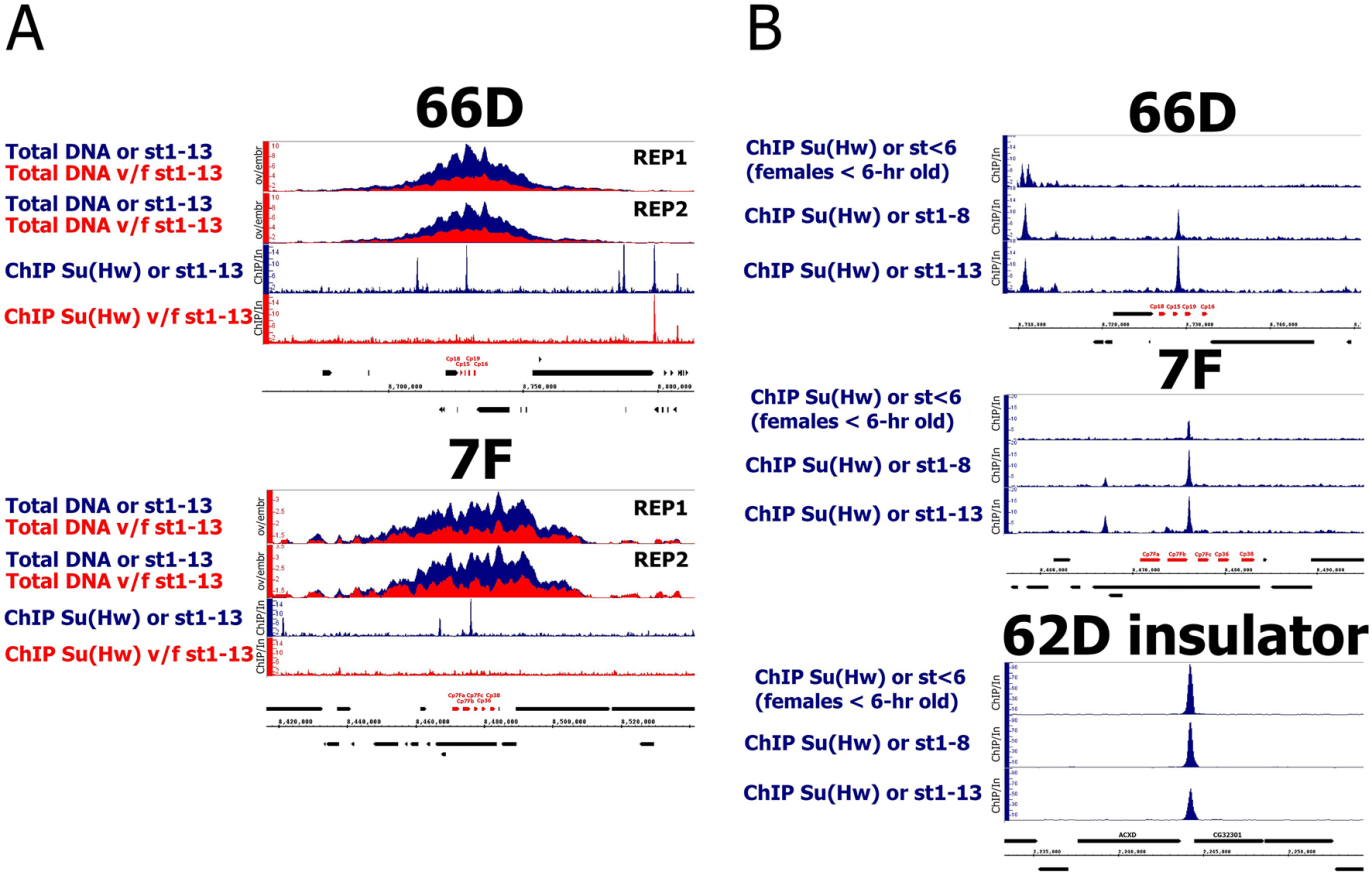
Su(Hw) is present in the 66D and 7F DAFCs and affects their amplification. (**A**) DNA-Seq and Su(Hw) binding profiles on 66D and 7F DAFCs in the wild type (or, blue plots) and *su(Hw)*^*v*/*f*^ (v/f, red plots) ovaries. Only the ovaries containing egg chambers stages 1–13 were selected for analysis. For DNA-Seqs two replicates of each sample are present (rep1, rep2). Su(Hw) binding level was estimated by ChIP-Seq and represent the enrichment of ChIP-Seq signal over input. The coordinates of the X-axis correspond to the dm6 version of the *Drosophila* genome. The coordinates of the Y-axis represent the (ovarian DNA/embryonic DNA) ratio for DNA-Seqs and the ChIP/Input ratio for ChIP-Seqs. The main chorionic genes of the loci are marked in red. (**B**) Su(Hw) binding profiles on 66D and 7F DAFCs and 62D insulator on three consecutive stages of the wild type (or) ovaries development: stages < 6 (females < 6-h old), stages 1–8 and stages 1–13 of the egg chambers. Su(Hw) binding level was estimated by ChIP-Seq and represent the enrichment of ChIP-Seq signal over input. The Su(Hw) ChIP-Seq for the stages < 6 (females < 6-h old) of egg chambers was taken from GSE86243 (described for the first time in^24^). The coordinates of the X-axis correspond to the dm6 version of the *Drosophila* genome. The coordinates of the Y-axis represent the ChIP/Input ratio. The main chorionic genes of the loci are marked in red.

It was previously demonstrated that Su(Hw) binding is retained at a portion of SBSs in *su(Hw)*^*v*/*f*^ mutant flies^23,24^. Therefore, we first tested whether Su(Hw) binding to its sites in DAFCs is altered in *su(Hw)*^*v*/*f*^ mutants using ChIP-Seq with antibodies against Su(Hw). For this analysis we dissected whole ovaries from 32 to 34 h-old wild-type (*oregon*) and *su(Hw)*^*v*/*f*^ mutant females. These ovaries already contain the stage 1–13 egg chambers but do not yet carry embryos^25^. We detected Su(Hw) binding at all known DAFCs in wild type; however, binding was only disrupted at the 66D and 7F loci in the *su(Hw)*^*v*/*f*^ background (Figs. 1A and S1). Therefore, we further focused on only the 66D and 7F DAFCs. To test the influence of Su(Hw) on amplification of the 66D and 7F loci, we carried out whole-genome sequencing of total DNA from the wild-type and *su(Hw)*^*v*/*f*^ ovaries. We observed a two-fold decrease in the amplification level of the 66D and 7F DAFCs upon *su(Hw)*^*v*/*f*^ mutation (Fig. 1A).

For this amplification analysis we used the whole ovaries, not just the stage 10–13 egg chambers. Therefore, the reduction in 66D and 7F amplification level we observed in *su(Hw)*^*v*/*f*^ may be due to a change in the ratio of stage 10–13 egg chambers in the ovaries of mutant flies. Indeed, an increase (10.5%) in apoptosis of mid-stage ovarioles was reported for *su(Hw)*^*v*/*f*^ ovaries in comparison to the wild type^22^. However, the observed Su(Hw)-dependent decrease in the amplification of 66D and 7F loci (Fig. 1A) significantly exceeds the changes expected due to the features of *su(Hw)*^*v*/*f*^ oogenesis described above. We suggest that the main reason underlying this decrease is the impact of Su(Hw) on the rate of amplification rather than an effect on the proportion of ovarioles containing amplified chorion loci in *su(Hw)*^*v*/*f*^ mutants.

To analyze the Su(Hw) effect on gene transcription in the 66D and 7F loci we isolated total RNA from wild-type and *su(Hw)*^*v*/*f*^ 32–34 h-old female ovaries and performed RNA-Seq. Surprisingly, we found no change in the transcription levels of the chorion genes located within the 66D and 7F DAFCs nor any abnormalities in eggshell phenotypes of *su(Hw)*^*v*/*f*^ embryos (Figs. S2, S3 and Table S1). However, as 66D and 7F amplification is two-fold reduced in *su(Hw)*^*v*/*f*^, the transcription of chorion genes relative to gene copy number is increased in *su(Hw)*^*v*/*f*^ ovaries compared to wild type.

### Pre-RC recruitment to the 66D and 7F chorion loci during oogenesis is impaired in ***su(Hw)***^***v/E8***^ mutants

We analyzed the role of Su(Hw) in the recruitment of replication factors during DAFCs amplification in the wild-type and *su(Hw)*^*v*/*f*^ ovaries containing 1–13 egg chamber stages. We performed ChIP-Seq experiments with antibodies against the ORC2 subunit of the origin recognition complex (ORC) and the CDC6 replication licensing protein. These proteins together with Double parked (Cdt1) participate in the loading of MCM2-7 replicative helicase and are known to form pre-RCs that are required for further initiation of DNA replication^26^. We detected a decrease of ORC2 and CDC6 binding in 66D and 7F DAFCs in *su(Hw)*^*v*/*f*^ ovaries compared to the wild type in ChIP-Seq samples prior to input normalization (Fig. S4). At the same time we also observed a decrease in the number of *su(Hw)*^*v*/*f*^ input reads of these loci, which we associate with overall reduced amplification in this background. After input normalization, the ORC2 and CDC6 profiles are similar between the wild-type and *su(Hw)*^*v*/*f*^ ovaries. As the levels of replication protein binding directly correlate with the level of amplification^19^, we assumed that the inputs and ORC2/CDC6 ChIP-Seq profiles in 66D and 7F DAFCs during amplification are dependent parameters. Therefore, we suggested that the effect of Su(Hw) on replication protein recruitment to DAFCs cannot be estimated correctly during DAFC amplification. Besides we admit the possibility that the wild-type and *su(Hw)*^*v*/*f*^ ovaries of 1–13 egg chamber stages may contain a slightly different ratio of egg chamber stages. It may potentially affect the amount of replication factors detected in ChIP-Seq analysis.

Previously we had shown that the ORC3 subunit of the ORC complex is recruited to SBSs in 66D and 7F loci in a Su(Hw)-dependent manner during the early stages of oogenesis. Therefore we hypothesized that the study of Su(Hw) impact on pre-RCs formation in DAFCs prior to their amplification might shed light on the subsequent effect on amplification. For this we used *su(Hw)*^*v*/*E8*^ heteroallelic mutant flies, which have a complete loss of Su(Hw) binding to chromatin^22^. Female flies carrying *su(Hw)*^*v*/*E8*^ alleles are sterile and suffer egg chamber degeneration at stage 9^21,22^. Therefore, for the analysis we dissected ovaries of recently eclosed 15 h-old flies, which contain egg chambers no older than stage 8^25^ (see Materials and Methods for details).

We analyzed Su(Hw) binding in wild-type and *su(Hw)*^*v*/*E8*^ ovaries in ChIP-Seq. As expected Su(Hw) binding was completely disrupted genome wide in the *su(Hw)*^*v*/*E8*^ ovaries (Fig. S5). Interestingly, we observed Su(Hw) binding to some SBSs changes during wild-type ovary development (Fig. 1B). SBSs in 66D and 7F DAFCs demonstrated an increase in Su(Hw) binding in the late stages of oogenesis, while binding at some other SBSs dropped. In total we observed that Su(Hw) binding at 242 SBSs is increased more than two-fold and 211 SBSs demonstrate greater than two-fold decrease during the transition from the early oogenesis (1–8 egg chamber stages) to late oogenesis (1–13 egg chamber stages) (Table S2). This observation calls into question the hypothesis that insulator proteins remain stably bound during development^24^.

**Figure 2:**
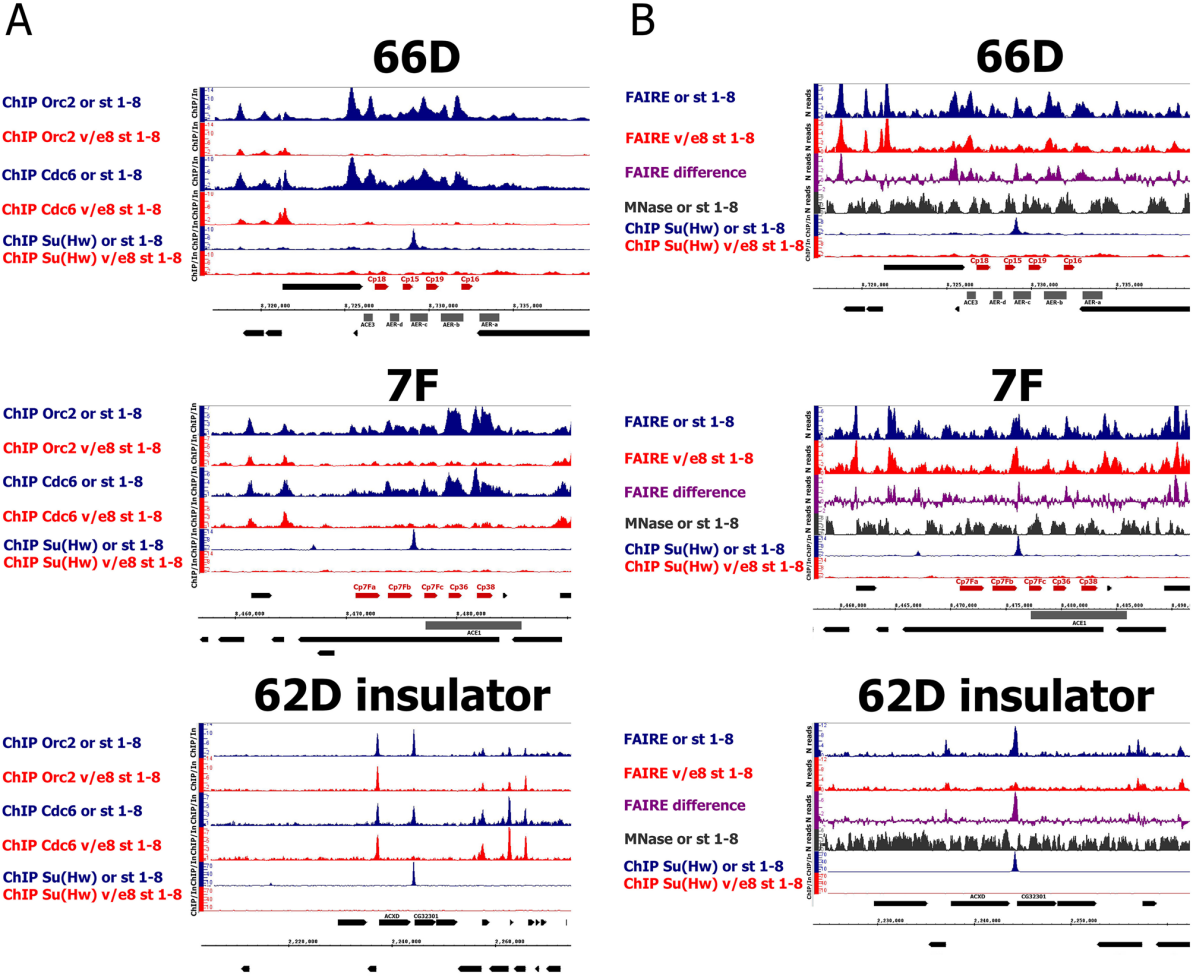
The absence of Su(Hw) in 66D and 7F DAFCs results in the depletion of replication proteins binding correlating with chromatin condensation at amplification regulatory regions of the loci during early oogenesis. (**A**) The binding profiles of ORC2 and CDC6 replication proteins on 66D and 7F DAFCs and on 62D insulator in the wild-type (or, blue plots) and *su(Hw)*^*v*/*E8*^ (v/e8, red plots) ovaries. Only the ovaries containing egg chambers stages 1–8 were selected for analysis. Additionally, the distributions of Su(Hw) in the wild-type (or, blue plots) and *su(Hw)*^*v*/*E8*^ (v/e8, red plots) ovaries are shown. Protein binding levels were estimated by ChIP-Seq and represent the enrichment of ChIP-Seq signal over input. The coordinates of the X-axis correspond to the dm6 version of the *Drosophila* genome. The coordinates of the Y-axis represent the ChIP/Input ratio. The main chorionic genes and amplification regulatory regions of the loci are marked in red and in grey, respectively. (**B**) The open chromatin profiles according to FAIRE-Seq enrichments on 66D and 7F DAFCs and on 62D insulator in the wild-type (or, blue plots) and *su(Hw)*^*v*/*E8*^ (v/e8, red plots) ovaries. Only the ovaries containing egg chambers stages 1–8 were selected for analysis. The difference between FAIRE-Seq profiles in the wild type and *su(Hw)*^*v*/*E8*^ ovaries ((FAIRE or 1–8)-(FAIRE v/e8 1–8)) is shown in violet plot. The MNase-Seq (NCBI-SRA BioProject SRP057811) for the follicular cells, extracted from the stages 1–8 of egg chambers, is shown in a black plot (described for the first time in^27^). Additionally, the distributions of Su(Hw) in the wild-type (or, blue plots) and *su(Hw)*^*v*/*E8*^ (v/e8, red plots) ovaries are shown. The coordinates of the X-axis correspond to the dm6 version of the *Drosophila* genome. The coordinates of the Y-axis represent the number of reads for FAIRE-Seqs and MNase-Seq and the ChIP/Input ratio for ChIP-Seqs. The main chorionic genes and amplification regulatory regions of the loci are marked in red and in grey, respectively.

We performed ChIP-Seq experiments with antibodies against the ORC2 and CDC6 replication proteins. In confirmation of previously published data^9^ we found that the recruitment of ORC2 and CDC6 to SBSs genome wide is disrupted in the *su(Hw)*^*v*/*E8*^ ovaries in comparison with wild type (Fig. S6). As a control we tested ORC2 and CDC6 recruitment to all known *Drosophila* transcription start sites (TSSs) and found no significant decrease in their binding between the wild-type and *su(Hw)*^*v*/*E8*^ ovaries (Fig. S6). The obtained whole-genome data allowed us to analyze the recruitment of ORC2 and CDC6 to 66D and 7F loci. We detected a strong decrease in binding of these replication proteins upon Su(Hw) mutation (Fig. 2A). Changes in ORC2 and CDC6 binding in *su(Hw)*^*v*/*E8*^ occurred at the positions of all known amplification regulatory regions of the loci^27^, and not only at SBSs. We attribute this effect to the impact of Su(Hw) on the chromatin structure of the loci as described below.

### Loss of chromatin-bound Su(Hw) in ***su(Hw)***^***v/E8***^ mutants leads to chromatin condensation at 66D and 7F loci during oogenesis

Pre-replication proteins lack apparent sequence specificity and are rather enriched at nucleosome-depleted and H3.3 marked regions^28–30^. We suggest chromatin condensation may be the primary reason for the disruption of pre-RC formation in *su(Hw)*^*v*/*E8*^ mutants. We performed FAIRE-Seq to compare chromatin state in wild-type and *su(Hw)*^*v*/*E8*^ mutant ovaries during stages 1–8 of egg chamber development. This method determines the profile of open chromatin and has proved to be an effective way to test nucleosome density at regulatory regions^31^.

We calculated the average distribution of FAIRE-Seq signal on all SBSs for the wild-type and *su(Hw)*^*v*/*E8*^ FAIRE-Seqs (Fig. S7). These data confirmed that SBSs are regions with an open chromatin structure, and the lack of Su(Hw) binding to SBSs in *su(Hw)*^*v*/*E8*^ mutants causes decreased chromatin accessibility in these regions. We detected broad regions of open chromatin encompassing the amplification regulatory regions from ACE3 to AER-A in 66D and at ACE1 in 7F in the wild-type ovaries (Fig. 2B). Our data are in good inverse correlation with MNase-Seq data of wild-type (*oregon*) ovaries of the same developmental stage^27^. The decondensed chromatin in 66D and 7F was found to significantly overlap with ORC2 and CDC6 binding sites from our ChIP-Seq experiments above (Fig. 2B). Furthermore, we observed a significant increase in nucleosome density in the amplification regulatory regions of 66D and 7F loci in the *su(Hw)*^*v*/*E8*^ background. Thus, the disruption of pre-RC binding coincides with chromatin condensation of these regions in *su(Hw)*^*v*/*E8*^ mutants.

### Chromatin condensation at the 66D and 7F loci in ***su(Hw)***^***v/E8***^ mutants cannot be attributed to a loss of Brm, ISWI, Mi-2, or CHD1 remodeler binding

**Figure 3:**
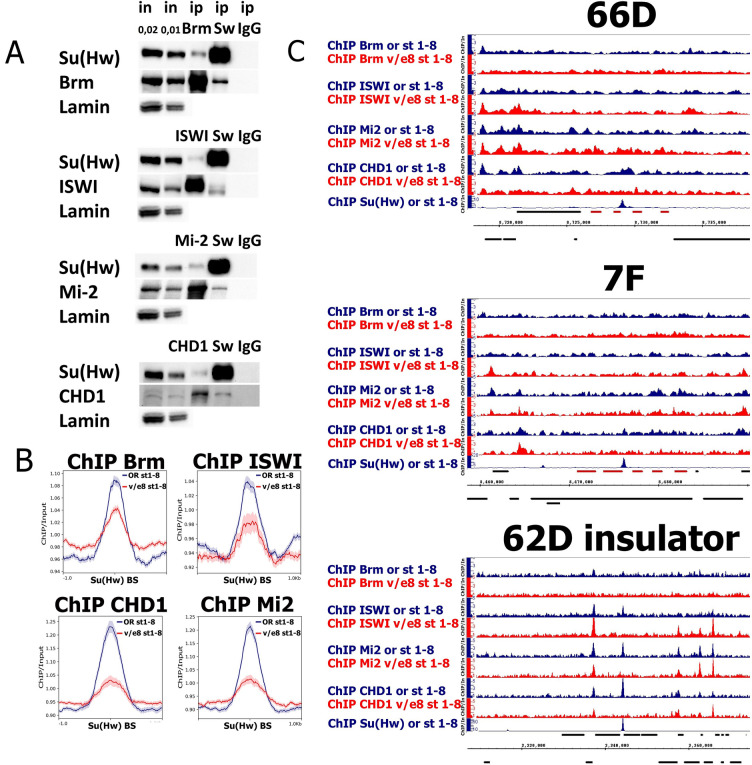
Su(Hw) recruits Brm, ISWI, Mi-2 and CHD-1 remodelers to SBSs but not in 66D and 7F DAFCs. (**A**) Immunoprecipitations (IPs) from nuclear protein extracts of *Drosophila* S2 cells. IPs were performed with antibodies against Brm, ISWI, Mi-2, CHD1 remodelers and Su(Hw) (a serum of non-immunized rabbits (ip IgG) was used as a negative control), which is indicated on the top of the figure. Western blots were stained with the corresponding antibodies indicated on the left of the figure. Anti-lamin staining was used as loading control. All input and IP samples were loaded on a single western blot. The original Western blots are present in Fig. S15. The numbers above the inputs represent a portion of a loaded fraction (in respect to the amount used for immunoprecipitations). (**B**) Average distribution of Brm, ISWI, Mi-2 and CHD1 remodelers binding on Su(Hw)-bound sites in the wild-type (or, blue profiles) and *su(Hw)*^*v*/*E8*^ (v/e8, red profiles) ovaries. The list of Su(Hw)-bound sites was calculated basing on our Su(Hw) ChIP-Seq data for the wild-type ovaries of stages 1–8. Brm, ISWI, Mi-2 and CHD1 binding levels were calculated as an enrichment (ratio of corresponding ChIP-Seq signal over input DNA). Average profiles were calculated as a median of Brm, ISWI, Mi-2 and CHD1 binding levels. The standard error is displayed on the graphs as lighter area around the main line of the profiles. (**C**) The binding profiles of Brm, ISWI, Mi-2 and CHD1 remodelers on 66D and 7F DAFCs and on 62D insulator in the wild-type (or, blue plots) and *su(Hw)*^*v*/*E8*^ (v/e8, red plots) ovaries. Only the ovaries containing egg chambers stages 1–8 were selected for analysis. Additionally, the distributions of Su(Hw) in the wild-type (or, blue plots) ovaries is shown. Protein binding levels were estimated by ChIP-Seq and represent the enrichment of ChIP-Seq signal over input. The coordinates of the X-axis correspond to the dm6 version of the *Drosophila* genome. The coordinates of the Y-axis represent the ChIP/Input ratio. The main chorionic genes of the loci are marked in red.

We hypothesized that chromatin condensation of the 66D and 7F loci in *su(Hw)*^*v*/*E8*^ mutant ovaries may be due to disrupted nucleosome remodeler recruitment. Earlier it was shown that SBSs overlap with published genome-wide binding datasets for a variety of proteins, including Brm and ISWI remodelers^12^. To define which remodelers could be affected by disrupted Su(Hw) binding, we tested Su(Hw) interaction with several ATP-dependent chromatin remodelers. We performed co-immunoprecipitations (co-IPs) with antibodies against Su(Hw) and reciprocal experiments with antibodies against representatives of the three chromatin remodeling families: Brahma (SWI/SNF family), ISWI (ISWI family), and Mi-2 and CHD1 (CHD family). Su(Hw) successfully co-precipitated all investigated remodelers from *Drosophila* S2 cell nuclear extracts (Fig. 3A). In the reciprocal experiments Brahma, ISWI, Mi-2, and CHD1 also co-precipitated Su(Hw), indicating that Su(Hw) can form complexes with these remodelers.

To investigate whether Brahma, ISWI, Mi-2, or CHD1 bind SBSs we performed ChIP-Seq. We detected enrichment of all investigated remodelers on SBSs in wild-type ovaries. This binding was disrupted in *su(Hw)*^*v*/*E8*^ ovaries, with the most significant perturbation in Mi-2 and CHD1 recruitment (Fig. 3B), while no significant difference in remodelers’ binding between wild-type and *su(Hw)*^*v*/*E8*^ ovaries was detected at the TSSs of *Drosophila* (Fig. S8). Intriguingly, when we evaluated the remodeler binding profiles within 66D and 7F DAFCs (Fig. 3C), we did not detect any significant remodeler-binding peaks colocalizing with SBSs or amplification regulatory regions in these loci. Thus, despite the fact that Su(Hw) recruits Brahma, ISWI, Mi-2, and CHD1 to SBSs in the ovaries, they are likely not responsible for the observed chromatin compaction at 66D and 7F DAFCs in the *su(Hw)*^*v*/*E8*^ mutant background.

The zinc-finger domain of Su(Hw) can participate in interactions with cofactor proteins^4,32^. Since Su(Hw) binding is retained at a portion of SBSs in *su(Hw)*^*v*/*f*^ mutant flies^23,24^, we tested whether the disruption of the tenth zinc finger in Su(Hw)^f^ (which is expressed in *su(Hw)*^*v*/*f*^) alters the ability of Su(Hw) to recruit remodelers to SBSs. We performed ChIP with antibodies against Brm, ISWI, Mi-2, and Chd1 on wild-type and *su(Hw)*^*v*/*f*^ ovaries containing 1–13 egg chamber stages (Fig. S9). While we observed a decrease in remodeler recruitment on SBSs with altered Su(Hw)^f^ binding compared to the wild-type, remodeler binding was not disrupted at *su(Hw)*^*v*/*f*^-retained SBSs. Thus we conclude Su(Hw)^f^ has the same ability to recruit remodelers as the wild-type Su(Hw) protein.

### Loss of chromatin-bound Su(Hw) causes the disruption of Gcn5 histone acetyltransferase binding to SBSs at the 66D and 7F loci

We hypothesized that chromatin condensation of amplification regulatory regions in *su(Hw)*^*v*/*E8*^ may occur due to the changes in histone acetylation at the 66D and 7F loci, as these modifications can affect chromatin state^33–35^. The CBP/Nejire and Chameau (Chm) histone acetyltransferases were previously demonstrated to be important for DAFCs amplification^36^, and Su(Hw) was shown to interact with the Gcn5 acetyltransferase to recruit pre-RCs to SBSs^9^. Thus we decided to analyze Gcn5 recruitment to the 66D and 7F DAFCs in wild-type and *su(Hw)*^*v*/*E8*^ ovaries.

**Figure 4:**
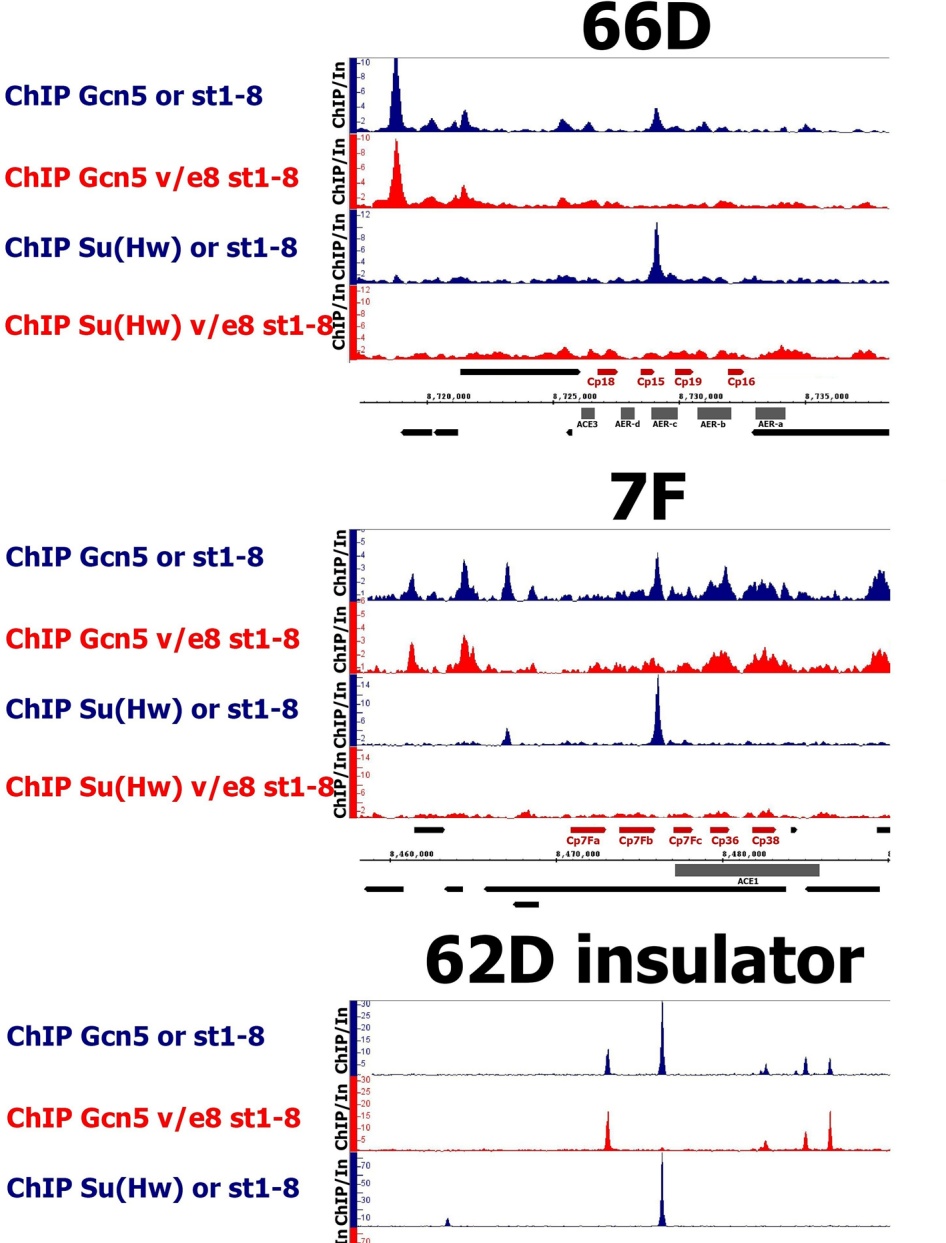
Gcn5 acetyltransferase is recruited to 66D and 7F loci in Su(Hw)-dependent manner. The binding profiles of Gcn5 acetyltransferase on 66D and 7F DAFCs and on 62D insulator in the wild-type (or, blue plots) and *su(Hw)*^*v*/*E8*^ (v/e8, red plots) ovaries. Only the ovaries containing egg chambers stages 1–8 were selected for analysis. Additionally, the distributions of Su(Hw) in the wild-type (or, blue plots) and *su(Hw)*^*v*/*E8*^ (v/e8, red plots) ovaries are shown. Protein binding levels were estimated by ChIP-Seq and represent the enrichment of ChIP-Seq signal over input. The coordinates of the X-axis correspond to the dm6 version of the *Drosophila* genome. The coordinates of the Y-axis represent the ChIP/Input ratio. The main chorionic genes and amplification regulatory regions of the loci are marked in red and in grey, respectively.

We performed ChIP-Seq with antibodies against Gcn5. As expected, Gcn5 was recruited to SBSs in the wild-type ovaries, and this recruitment was disrupted in *su(Hw)*^*v*/*E8*^ mutant ovaries (Fig. S10). As a control we tested Gcn5 recruitment to all annotated *Drosophila* TSSs and found no significant difference in binding between wild-type and *su(Hw)*^*v*/*E8*^ ovaries. Within the central regions of the 66D and 7F loci we detected several Gcn5 peaks, with the highest peaks colocalizing with SBSs (Fig. 4). In *su(Hw)*^*v*/*E8*^ mutants we observed a total disruption of Gcn5 binding at SBSs within 66D and 7F. Thus, Su(Hw)-dependent recruitment of Gcn5 to 66D and 7F DAFCs suggests that, in addition to CBP/Nejire and Chm, Gcn5 can regulate developmental amplification of DAFCs.

We then performed Gcn5 ChIP on the wild-type and *su(Hw)*^*v*/*f*^ ovaries containing 1–13 egg chamber stages to determine whether Su(Hw)^f^ (expressed in *su(Hw)*^*v*/*f*^) can recruit Gcn5 (Fig. S9). Gcn5 recruitment to *su(Hw)*^*v*/*f*^-retained SBSs was not affected, which suggests Su(Hw)^f^ has the same ability to recruit Gcn5 as the wild-type Su(Hw).

### Clustering analysis confirms the existence of replicational SBS subclass

**Figure 5:**
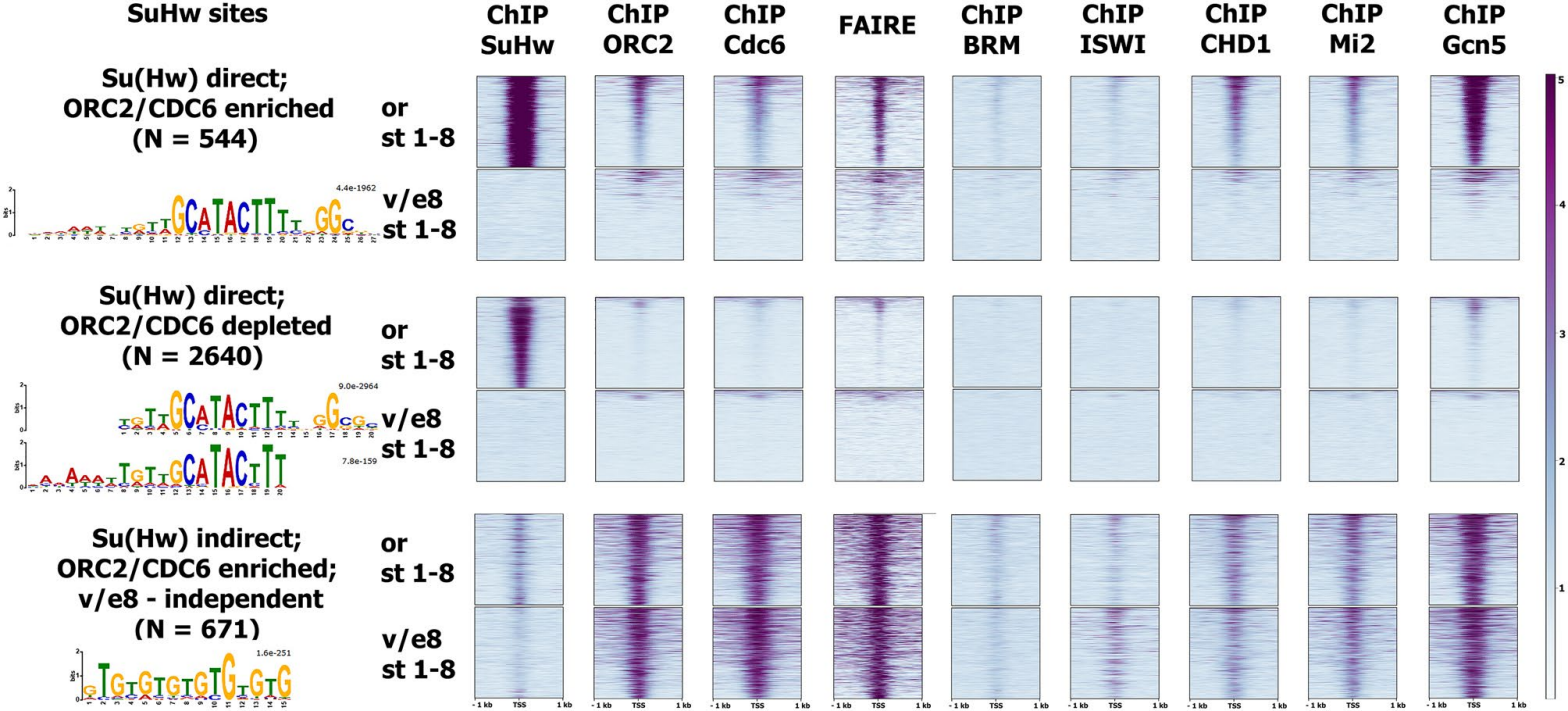
Clustering analysis confirms the existence of replicational SBS subclass. Heatmap showing ChIP-Seq signals for various proteins or FAIRE-Seq signal surrounding cluster 1 (“Su(Hw) direct; ORC2/CDC6 enriched”), cluster 2 (“Su(Hw) direct; ORC2/CDC6 depleted”) and cluster 3 (“Su(Hw) indirect; ORC2/CDC6 enriched; v/e8-independent”) SBSs ± 1 kb in in the wild-type and *su(Hw)*^*v*/*E8*^ ovaries containing egg chambers stages 1–8. MEME-generated consensus sequences for the cluster 1–3 SBSs are shown on the left of the heatmap (E-values for corresponding motifs are indicated).

SBSs can be divided into three subclasses based on distinct motif characteristics: insulator (M4-only), replicational (M4 + M10), and transcriptional repressor (M10-only)^12^. We used our ChIP-Seq and FAIRE-Seq datasets for the wild-type and *su(Hw)*^*v*/*e8*^ ovaries to assign SBSs into functional groups by performing k-means clustering (Fig. 5). SBSs split into 3 clusters with different characteristics. Cluster 1 is comprised of the 544 strongest SBSs with high levels of ORC2 and CDC6 binding, which is completely disrupted in *su(Hw)*^*v*/*e8*^ (“Su(Hw) direct; ORC2/CDC6 enriched”). This cluster is responsible for the majority of Su(Hw)-dependent chromatin remodeler and Gcn5 recruitment to SBSs and for chromatin opening. Cluster 2 includes 2640 SBSs devoid of ORC2/CDC6 binding and located in condensed chromatin regions (“Su(Hw) direct; ORC2/CDC6 depleted”). Cluster 3 includes weak Su(Hw)-bound sites located in accessible chromatin and highly enriched with ORC2/CDC6. Su(Hw) and other factors remain bound at cluster 3 sites in *su(Hw)*^*v*/*e8*^ (“Su(Hw) indirect; ORC2/ CDC6 enriched; *su(Hw)*^*v*/*e8*^-independent”). It is known that *su(Hw)*^*v*/*E8*^ mutants express Su(Hw) protein completely unable to bind chromatin^22^. We believe this cluster includes Su(Hw) peaks arising from indirect Su(Hw) recruitment mediated by interactions with other DNA-binding proteins. Thus, the recruitment of replication proteins to the sites of cluster 3 is likely determined by other factors.

To link the identified SBS clusters to known Su(Hw) motifs, we carried out motif analysis with the MEME motif search program (Fig. 5). This analysis confirmed that cluster 1 SBSs are characterized with M4 + M10 SBS subclass motif, which has previously been associated replicational functions^12^. Cluster 2 demonstrated the enrichment with either M4- and M10-only motifs associated with Su(Hw) insulator and transcriptional repressor functions. We were unable to identify a Su(Hw) motif in cluster 3 sequences but rather found that this cluster is enriched with a GTGT-motif.

We then classified previously defined SBSs with Su(Hw) binding, which changes during the wild-type ovary development, according to the SBS clusters (Table S2 and Fig. S11). We observed that the majority of these SBSs do not overlap with SBS cluster 1, associated with replicational functions. SBSs with Su(Hw) binding increased during oogenesis (240 out of 242) correspond to cluster 2 and are enriched with M10-only motif, associated with Su(Hw) transcriptional repressor function. SBSs with Su(Hw) binding decreased during oogenesis (164 out of 211) mostly identify as cluster 3 SBSs. They contain the GTGT-motif characteristic of this cluster and GA repeats resembling the motifs of several DNA binding proteins^37,38^ (Fig. S11). Therefore, transcription regulation during development appears to be a more dynamic function of Su(Hw) than the regulation of replication.

## Discussion

**Figure 6:**
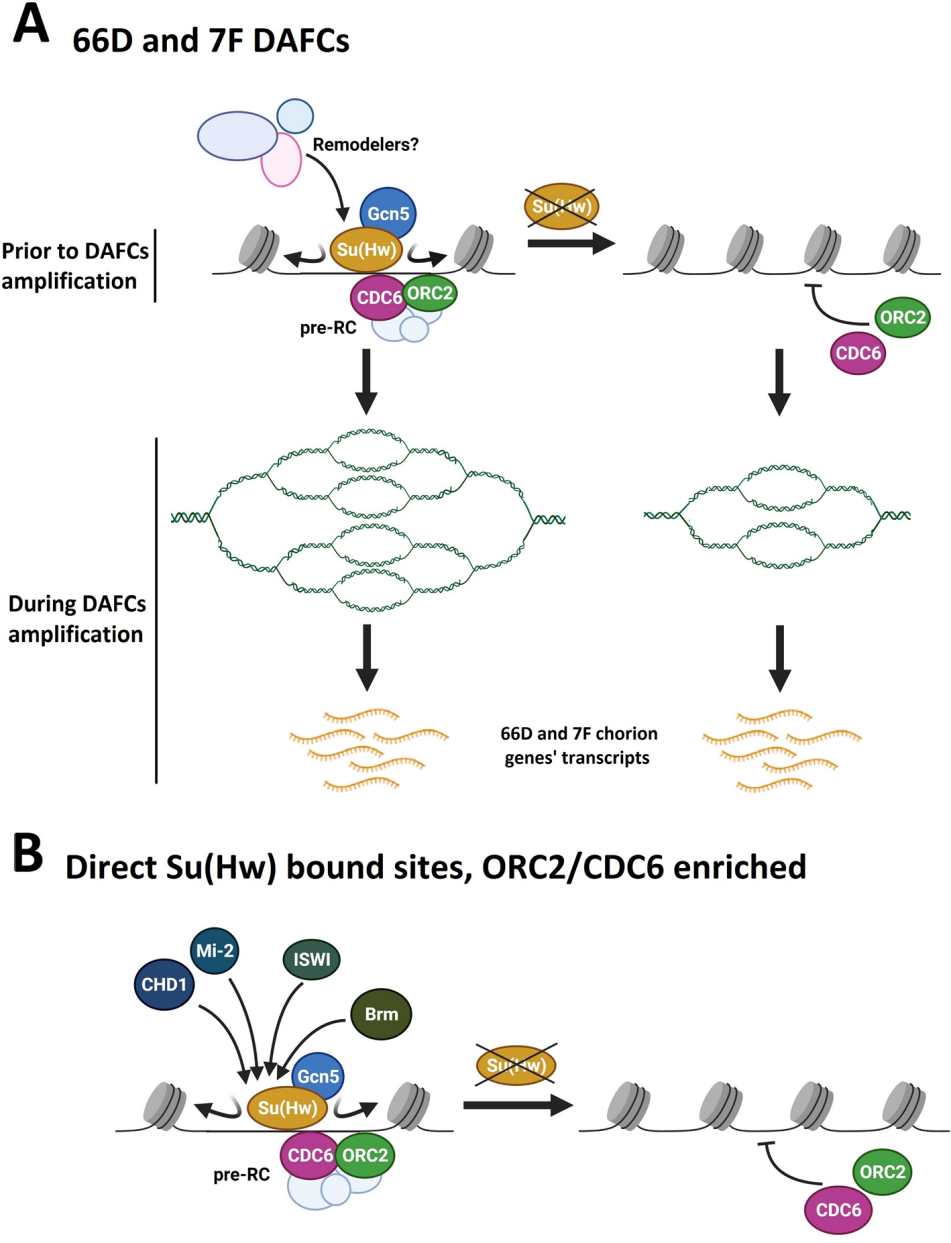
Proposed model describing a role of Su(Hw) in regulation of replication during oogenesis. (**A**) Proposed model describing a role of Su(Hw) in regulation of 66D and 7F DAFCs developmental amplification. Prior DAFCs amplification, Su(Hw) recruits Gcn5 acetyltransferase and organizes the open chromatin structure on amplification regulatory regions of these loci, which promotes the formation of pre-replication complexes (pre-RCs), containing ORC2 and CDC6. Its depletion results in the condensation of chromatin at amplification regulatory regions of 66D and 7F and the disruption of pre-RCs formation. At the late stages of oogenesis, the absence of Su(Hw) in 66D and 7F DAFCs results in the decrease in the amplification of these loci with no change in the transcription levels of the chorion genes located within the 66D and 7F DAFCs. (Image created using BioRender.com). (**B**) Extended model of Su(Hw) functioning at direct Su(Hw) bound sites, ORC2/CDC6 enriched in the genome of *Drosophila*. Su(Hw) recruits Gcn5 acetyltransferase and Brm, ISWI, Mi-2 and CHD1 remodelers on SBSs and organizes the regions with low nucleosome density. These regions serve as platforms for pre-RCs formation. (Image created using BioRender.com).

In spite of a long history of research on *Drosophila* Su(Hw) insulator protein its functions remain unclear. This is exemplified by the fact that many Su(Hw) binding sites in the genome do not correspond to its well-known repressive and enhancer-blocking activities^12^. Previously we described the ability of Su(Hw) to organize regions with low nucleosome density, which could serve as platforms for pre-RC binding in *Drosophila* S2 cells and pupa^9,10^. Here, we demonstrate a functional impact of Su(Hw) on the developmentally regulated amplification of the 66D and 7F *Drosophila* follicle cell amplicons (summarized at Fig. 6A).

### Su(Hw) is important for the amplification of 66D and 7F chorion genes loci

The high colocalization of Su(Hw) peaks with all DAFCs (Figs. 1A and S1) suggested the importance of Su(Hw) for DAFCs function. This was corroborated by our data showing that loss of Su(Hw) binding at the 66D and 7F loci resulted in a two-fold decrease in their amplification (Fig. 1A). To our knowledge, this is the first demonstration of Su(Hw) impact on replication.

Interestingly, the decrease in 66D and 7F DAFCs amplification rate in the *su(Hw)*^*v*/*f*^ mutant does not lead to a decrease in the transcription level of genes within these loci (Fig. S2). Instead, transcription of chorion genes relative to gene copy number is increased in *su(Hw)*^*v*/*f*^ ovaries compared to the wild type. This may be attributed to the know repressor role of Su(Hw), or might be the result of a Su(Hw)-independent compensation of chorion gene transcription during 66D and 7F loci underreplication. Further studies will be required to distinguish between these possibilities. The direct correlation between amplification of DAFCs and their transcription had already previously been called into question^19,20^, as well the potential role of local transcription in DAFCs amplification^39^. In line with this, our work suggests that the regulation of developmental gene amplification and transcription can be implemented through various mechanisms.

### Su(Hw) regulates initial pre-RC recruitment to the 66D and 7F loci

We analyzed ORC2 and CDC6 replication protein recruitment to 66D and 7F DAFCs at stages 1–8 of egg chamber development, prior to the start of DAFCs amplification. These proteins participate in the formation of pre-RCs, and are required for further initiation of DNA replication^26^. Previously it was thought that binding of replication proteins to DAFCs occurs at stage 10 of egg chamber development when the DAFCs amplification begins^27,40^; however, these conclusions were based on immunofluorescence analysis, which is not sensitive enough to detect low-level binding. By employing ChIP-Seq, we showed here that the recruitment of ORC2 and CDC6 to the amplification regulatory regions of 66D and 7F DAFCs begins already during early oogenesis at stages 1–8 of egg chamber development (Fig. 2A).

The complete loss of Su(Hw) binding in the *su(Hw)*^*v*/*E8*^ background impairs ORC2 and CDC6 recruitment to 66D and 7F. Intriguingly, the disruption of replication proteins recruitment occurs not only at SBSs within these loci but at all amplification regulatory regions. It is well-established that replication proteins lack apparent sequence specificity but are rather enriched at nucleosome-depleted regions^28^. This led us to attribute the loss of ORC2 and CDC6 binding within the 66D and 7F loci in *su(Hw)*^*v*/*E8*^ mutants to a potential Su(Hw)-dependent regulation of chromatin structure at these loci.

### Su(Hw) participates in the organization of open chromatin regions in 66D and 7F loci

In our previous work, we showed Su(Hw) depletion increases chromatin compaction^9,10^. We therefore reasoned that changes in chromatin accessibility may underlie the Su(Hw)-dependent disruption of pre-RC formation in 66D and 7F loci. We detected open chromatin regions in the 66D and 7F loci in wild-type ovaries by FAIRE-Seq, which coincided with amplification regulatory elements of these loci (Fig. 2B). A loss of Su(Hw) binding in *su(Hw)*^*v*/*E8*^ mutants led to a strong decrease in the chromatin accessibility of 66D and 7F at regions of ORC2- and CDC6-binding.

To determine whether these effects on chromatin accessibility are caused by disrupted recruitment of chromatin-modifying complexes, we first determined that Su(Hw) interacts with the Brm, ISWI, Mi-2, and CHD1 remodelers by co-IP (Fig. 3A). Surprisingly, however, these remodelers are not present at the 66D and 7F loci (Fig. 3C). It can be suggested that chromatin remodeling by Brm, ISWI, Mi-2 and CHD1 is not the main factor determining the chromatin structure of 66D and 7F loci.

### Gcn5 acetyltransferase is recruited to 66D and 7F DAFCs in a Su(Hw)-dependent manner

Histone acetylation is known to affect the global chromatin state by weakening nucleosome-DNA interactions^33–35^. Histone H3 and H4 acetylation is required for chromatin decompaction during DNA replication^41,42^ and can regulate the time of origin firing^43^. Furthermore, the level of histone acetylation correlates with the amplification rate of DAFCs^44,45^. The histone acetyltransferases CBP/Nejire and Chm had previously been shown to bind to the 66D and 7F DAFCs and were found to be necessary for their normal amplification^36^. In the current work we showed that Gcn5 is yet another histone acetyltransferase present at these loci. Moreover, we identified Su(Hw) as the main recruiter of Gcn5 to these loci (Fig. 4). Gcn5 binding in 66D and 7F does not overlap with the Brm, ISWI, Mi-2, or CHD1 remodelers, implying that its function in the loci may be independent of chromatin remodeling.

Gcn5 has long been known as a positive regulator of genome DNA replication and a determinant of replication origin firing timing in yeast^43,46^. Recently it was reported that CHAT and SAGA GCN5-containing complexes mediate H3K14 acetylation at amplified chorion genes loci in *Drosophila* follicular cells^47^. Although the authors claimed that CHAT and SAGA are not essential for chorion genes amplification, their lack of quantitative measurements does not preclude a conclusion that Gcn5-containing complexes can indeed promote amplification at the loci to a certain level.

Apart from histone acetylation, Gcn5 can regulate replication by influencing replication licensing factors, which ensure that chromosomes are replicated once per cell cycle^48^. During G1 cyclin E phosphorylates the CDC6 replication protein to prevents its degradation and opens a “window of opportunity” for pre-RCs formation^49^. Upon entry into S-phase GCN5 acetylates CDC6, causing its release from chromatin and phosphorylation by cyclin A, which subsequently leads to its degradation^50^. These events prevent a second round of pre-RC formation. At the same time Gcn5-dependent acetylation of cyclin A promotes its degradation to determine the fate of germline stem cells in *Drosophila* ovaries^51^. Furthermore, a recent mass spectrometry-based proteomic screening identified the MCM2-7 replicative helicase complex as a putative Gcn5 acetylation substrate^52^. Given its myriad functions, future investigations should aim to delineate the specific role of Gcn5 during amplification of the 66D and 7F DAFCs.

### Su(Hw)-dependent recruitment of nucleosome remodelers and the Gcn5 acetyltransferase on SBSs in the ovaries correlates with chromatin decompaction and pre-RC formation

Our study significantly expands the existing model of Su(Hw) function in positioning pre-RCs on SBSs in the genome (Fig. 6B). Our ChIP-Seq data confirmed the recruitment of Gcn5 and the Brm subunit of the SWI/SNF remodeler family to SBSs, and also demonstrated that three additional remodelers, ISWI, Mi-2, and CHD1, are recruited to SBSs in a Su(Hw)-dependent manner (Figs. S10 and 3B). FAIRE-Seq demonstrated that Su(Hw) is responsible for open chromatin regions at SBSs in the ovary (Fig. S7). As the recruitment of remodelers is disrupted in *su(Hw)*-mutant background we suggest that they are involved in the formation of open chromatin regions on SBSs in the *Drosophila* ovary.

We also confirmed that loss of Su(Hw) causes genome-wide disruption of ORC2 and CDC6 recruitment to SBSs (Fig. S6). In a recent study it was shown that loss of Su(Hw) in egg chambers results in the accumulation of unrepaired DNA breaks associated with chromatin markers of replication stress^53^. We hypothesize that this impact on genome stability may indeed be due to disrupted pre-RC formation at SBSs in the ovary. Further investigations, such as ChIP-Seq data for γH2Av, a marker of double-stranded DNA breaks^54^, and H4K20me1, which correlates with replication stress^55^, in the ovaries of wild-type and *su(Hw)*-mutant flies will help to directly link the described role of Su(Hw) in genome integrity and our data on Su(Hw)-dependent pre-RC formation.

Clustering analysis based on our ChIP-Seq and FAIRE-Seq data identified the properties of SBS functional subclasses, which were predicted in^12^ (Fig. 5). In our analysis we identified three SBS clusters with distinct protein binding and motif characteristics. We observed that cluster 1 SBSs are associated with Su(Hw)-dependent chromatin opening and recruitment of replication proteins. According to motif analysis, our cluster 1 SBSs correspond to the M4 + M10 SBS subclass in^12^. Cluster 2 presents SBSs which do not demonstrate enrichment in replication proteins, remodelers, or Gcn5, and are located in condensed chromatin regions. Motifs identified for this cluster correlate well with the M4- and M10-only SBS subclass motifs. In addition to these classes of SBSs we identified a group of indirect Su(Hw)-bound sites (cluster 3) enriched with a GTGT-motif. This motif was previously linked with Combgap binding, which is involved in recruitment of PcG group proteins^56,57^. We propose this cluster reflects Su(Hw) indirect recruitment. Indirect binding was previously shown for BEAF32 architectural protein in ChIP-Seq analysis^58^. Basing on the GTGT-motif enrichment of cluster 3 sites we suggest that Su(Hw) recruitment to these sites may be mediated by interaction with Combgap protein. There is some evidence of a functional connection between Su(Hw) and Combgap. The ability of SBS to enhance the activity of Combgap-bound *bxd* Polycomb response element (PRE) in transgenic assay has recently been reported^59^. Analyzing our data we found *bxd* PRE among the cluster 3 Su(Hw) bound sites. Upon close examination of Su(Hw) ChIP-Seq profiles at the BX-C locus we did observe Su(Hw) binding at the *bxd* PRE which is independent of *su(Hw)*^*v*/*E8*^ background (Fig. S12). We believe that future research may shed light on Su(Hw) interaction with Polycomb group proteins.

In summary, our data suggest a new role for Su(Hw) in *Drosophila* oogenesis as a regulator of developmentally controlled 66D and 7F DAFCs amplification and contribute to a more comprehensive picture of Su(Hw) function in *Drosophila* development.

## Materials and methods

### Collection of the *Drosophila* ovaries of different developmental stage

The flies of Oregon-R-modENCODE (*oregon*) (corresponds to Bloomington stock 25,211), *su(Hw)*^*v*/*E8*^ and *su(Hw)*^*v*/*f*^ stocks (a kind gift of A. Golovnin laboratory) were used. All experiments on *Drosophila* reported in the manuscript follow the recommendations in the ARRIVE guidelines. All flies were raised at 25 °C on standard agar medium. Ovaries were dissected from two developmental groups of females: 15 h after eclosion (include egg chamber stages 1–8, Fig. S13) and 32–34 h after eclosion (include egg chamber 1–13). During dissection, we thoroughly controlled the correctness of the stages of egg chambers in the ovaries, collected for the analysis.

For the ChIP-Seq experiments ovaries were collected in PBS buffer (50 pairs per ChIP), fixed with 1% of formaldehyde for 5 min and then incubated for 5 min with 125 mM Glycine. Then ovaries were washed with PBS buffer for three times. The remaining ChIP protocol was performed as described previously^9^.

### ChIP, ChIP-Seq

The chromatin immunoprecipitation (ChIP) was performed and analyzed exactly as previously described^9,60^. ChIP-Seq libraries were obtained using the NEBNext Ultra™ II DNA library preparation kit (New England Biolabs). Only the library fragments of 250–500 bp were subjected to NGS sequencing. New generation sequencing was performed by Evrogen (evrogen.ru) with the Illumina NovaSeq6000 sequencer. For each of the ChIP‐Seq libraries approximately 5–12 millions of unique paired-end reads were obtained. The paired‐end reads in FastQ format were mapped to the *Drosophila* genome assembly dm6 using Bowtie2^61^ and filtered (with minimum MAPQ quality score = 5).

BigWig files were generated using bamCoverage 3.0.2 with scores representing number of reads normalized by the size of the library (the protein binding levels were normalized to the genome content—calculated as RPGC: number of reads per bin/(total number of mapped reads * fragment length/effective genome size)^62^. The final bigwig files (representing the protein binding profiles) were obtained using bigwigcompare tool as ratio of ChIP signal to Input (all inputs and ORC2/CDC6 ChIP samples for the wild-type and *su(Hw)*^*v*/*f*^ ovaries of egg chamber stages 1–13 were preliminary smoothed over a 1 kb window). Pile-up profiles were calculated as a median level of protein binding for the distributions of Su(Hw), ORC2, CDC6, Brm, ISWI, Mi-2, CHD1 and Gcn5 on Su(Hw) binding sites (SBSs) and for ORC2, CDC6, Brm, ISWI, Mi-2, CHD1 and Gcn5 on TSSs in the wild-type (*oregon*), *su(Hw)*^*v*/*E8*^ and *su(Hw)*^*v*/*f*^ ovaries of *Drosophila*. The SBSs in the wild type (*oregon*) ovaries (stages 1–8 of egg chamber development) were defined by MACS2 with the following parameters: -gsize '120,000,000' -keep-dup '1'-qvalue '0.01' -mfold '5' '30' --bw '375′ 2 > &1 > macs2_stderr^63^. Corresponding input DNA was used as a control for peak calling.

Clustering analysis was performed using plotHeatmap tool of deepTools package^62^. K-means clustering algorithm was selected and the number of clusters to compute was set to 3. The ChIP-Seq data were clustered using the values at the summits of the SBSs.

The definition of motifs for SBS cluster 1–3 was performed with MEME suite 5.3.3^64^.

The Galaxy-P platform was used for analysis of ChIP-Seq data^65^. All obtained ChIP-Seq data were deposited into the Gene Expression Omnibus—GSE168894.

MNase-Seq data from follicle cells (stages 1–8 of egg chamber development) of Oregon-R-modENCODE fly stock were described previously^27^ and deposited as NCBI-SRA BioProject SRP057811. We did not use any figures or text from the previously published manuscripts—only data deposited in free access databases.

### DNA-Seq, RNA-Seq

For DNA extraction the ovaries of 32–34 h old wild-type (*oregon*) and *su(Hw)*^*v*/*f*^ females were collected in PBS buffer (50 pairs per sample), in two biological repeats. Total DNA was extracted using ExtractDNA Blood kit (Evrogen). The DNA from 2 to 4 h old *Drosophila* embryos was extracted and used as non-amplified control. All obtained DNA was sheared to 250–500 bp by sonication. DNA-Seq libraries were obtained using the NEBNext Ultra™ II DNA library preparation kit (New England Biolabs). New generation sequencing was performed by Evrogen (evrogen.ru) with the Illumina NovaSeq6000 sequencer. For each DNA‐Seq library approximately 40–55 millions of unique paired-end reads were obtained (10 million paired-end reads were obtained for 2–4 h old embryo DNA). The paired‐end reads in FastQ format were mapped to the *Drosophila* genome assembly dm6 using Bowtie2^61^ and filtered (with minimum MAPQ quality score = 5). The final bigwig files (representing the ovarian DNA enrichment over embryonic DNA) were obtained using bigwigcompare tool as ratio of corresponding DNA from the ovaries to DNA from the 2–4 h old embryos (all samples were preliminary smoothed over a 2 kb window).

For extraction of RNA the ovaries of 32–34 h old wild-type (*oregon*) and *su(Hw)*^*v*/*f*^ females were collected in PBS buffer (50 pairs per sample), in two biological repeats. Total RNA was extracted with the TRI reagent (Ambion). PolyA comprising RNA fraction was isolated and prepared for sequencing with the NEBNext Ultra™ II Directional RNA Library Prep Kit. New generation sequencing was performed by Evrogen (evrogen.ru) with the Illumina NovaSeq6000 sequencer. For each RNA‐Seq library approximately 25–30 millions of unique reads were obtained. Obtained reads were mapped using TopHat2^66^. Differential analysis was employed by CuffDiff^67^. Obtained RNA-Seq data were deposited into the Gene Expression Omnibus—GSE168894.

### FAIRE-Seq

For FAIRE experiment the ovaries of 15 h old wild-type (*oregon*) and *su(Hw)*^*v*/*E8*^ females were collected in PBS buffer (50 pairs per sample). The FAIRE protocol was performed as previously described^31^. FAIRE-Seq libraries were obtained using the NEBNext DNA library preparation kit (New England Biolabs). Only the library fragments of 250–500 bp were subjected to NGS sequencing. New generation sequencing was performed by Evrogen (evrogen.ru) with the Illumina NovaSeq6000 sequencer. For each FAIRE‐Seq library, approximately 4–5 millions of unique paired-end mappable reads were obtained. The paired‐end reads in FastQ format were mapped to the *Drosophila* genome assembly dm6 using Bowtie2^61^ and filtered (with minimum MAPQ quality score = 5). Obtained FAIRE-Seq data were deposited into the Gene Expression Omnibus—GSE168894.

### Nuclear protein extract and co-immunoprecipitation

*Drosophila* Schneider cell line 2 (S2) cells were used for nuclear protein extract preparation. S2 cells were maintained at 25 °C in Schneider’s insect medium (Sigma) containing 10% FBS (HyClone). The nuclear protein extract was obtained as described previously^68^. Co-immunoprecipitations were performed as previously described^68^. The RNA-interference experiments to test antibodies specificity were performed as previously described^69^.

### Antibodies

For the co-immunoprecipitations and ChIP-Seq analysis of Brm, ISWI, Mi-2, CHD1 and GCN5 we used previously described antibodies^9,69^. Antibodies against Su(Hw) (epitope corresponding to the N-terminal domain), ORC2 (epitope corresponding to 28–194 amino acid residues) and CDC6 (epitope corresponding to 9–179 amino acid residues) were generated in this study. The epitopes for antibody production were expressed as 6 × His-tagged fusion proteins in *Escherichia coli*, affinity-purified on Ni Sepharose 6 Fast Flow (GE Healthcare), according to the manufacturer’s protocol, and injected into rabbits, following the standard immunization procedure. Antibodies were affinity-purified using the same epitopes as were used for immunization. The specificity of antibodies against Su(Hw), ORC2 and CDC6 was characterized in the RNA interference experiments by the specific depletion of corresponding proteins (Fig. S14). Antibodies production was performed according to procedures outlined in the NIH (USA) Guide for the Care and Use of Laboratory Animals and following the recommendations in the ARRIVE guidelines. The protocol used was approved by the Committee on Bioethics of the Institute of Gene Biology of the Russian Academy of Sciences. All procedures were performed under conditions designed to minimize suffering.

## Supplementary Information

Supplementary Information 1.
