# Supplementary material for: Su(Hw) primes 66D and 7F Drosophila chorion genes loci for amplification through chromatin decondensation
Source: Sci Rep. 2021 Aug 20;11:16963. doi: 10.1038/s41598-021-96488-0 (PMC8379230; doi:10.1038/s41598-021-96488-0)
Supplement: Supplementary file 1 — Supplementary Information 1. [file 41598_2021_96488_MOESM1_ESM.pdf]

Su(Hw) primes 66D and 7F *Drosophila* chorion genes loci for amplification through chromatin decondensation

Nadezhda E. Vorobyeva[1], Maksim Erokhin[1], Darya Chetverina[1], Alexey N. Krasnov[1**], Marina Yu. Mazina[1*]

[1]Institute of Gene Biology, Russian Academy of Sciences, Moscow 119334, Russia

* Corresponding author (email to mazinam@genebiology.ru)

** Corresponding author (email to krasnov@genebiology.ru)

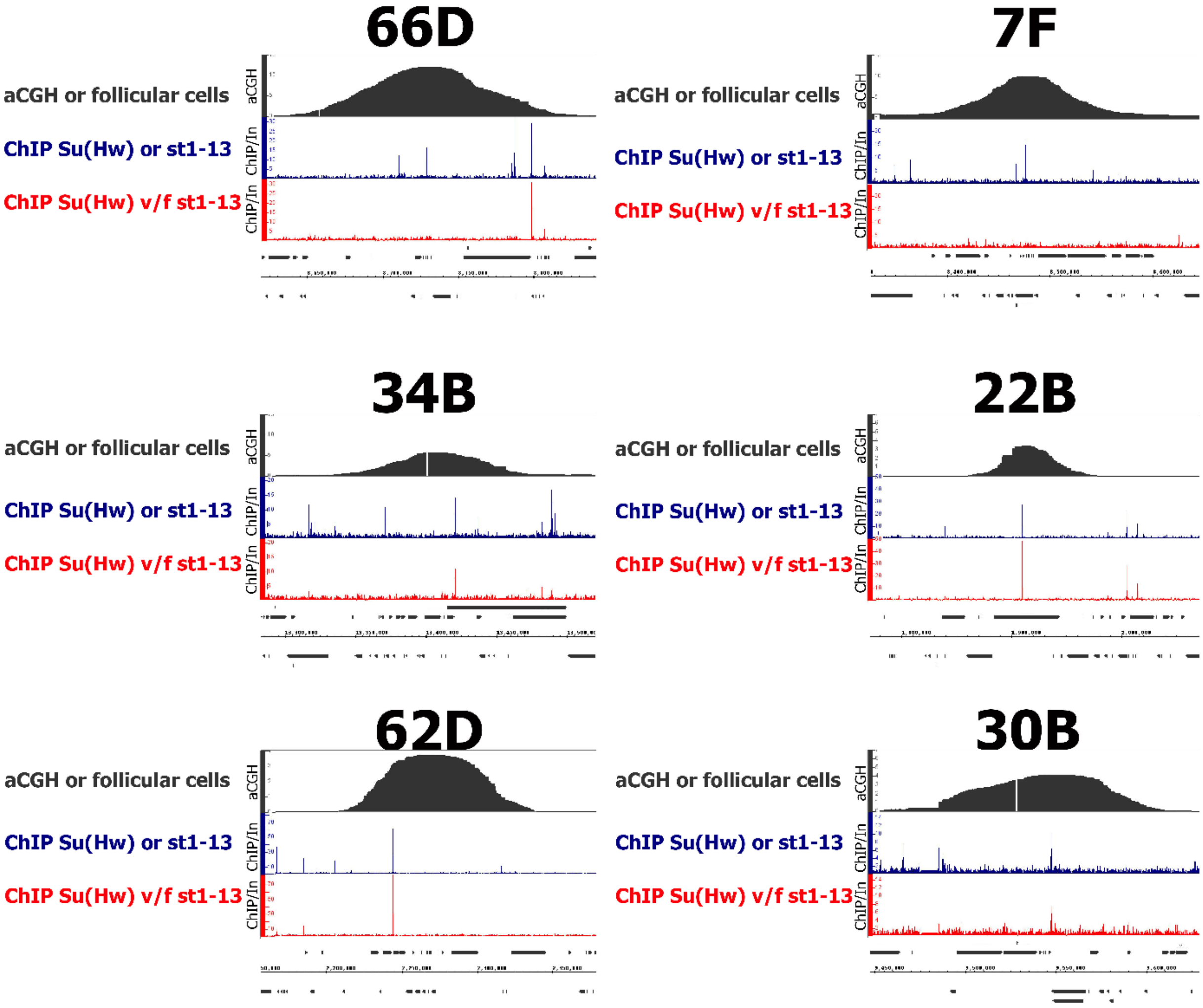

Supplementary figure 1. Su(Hw) binding profiles on 34B, 22B, 62D and 30B DAFCs in the wild type (or, blue profiles) and *su(Hw)*$^{v/f}$ (v/f, red profiles) ovaries. Array-based comparative genomic hybridization (aCGH) profiles for follicle cells of egg chamber stage 10 of the wild type ovaries (or, black) are also present (described for the first time in [18]). Only the ovaries containing egg chambers stages 1-13 were selected for analysis. Su(Hw) binding level was estimated by ChIP-Seq and represent the enrichment of ChIP-Seq signal over input. The coordinates of the X-axis correspond to the dm6 version of the *Drosophila* genome. The coordinates of the Y-axis represent the (ovarian DNA/embryonic DNA) ratio for DNA-Seq and the ChIP/Input ratio for ChIP-Seqs.

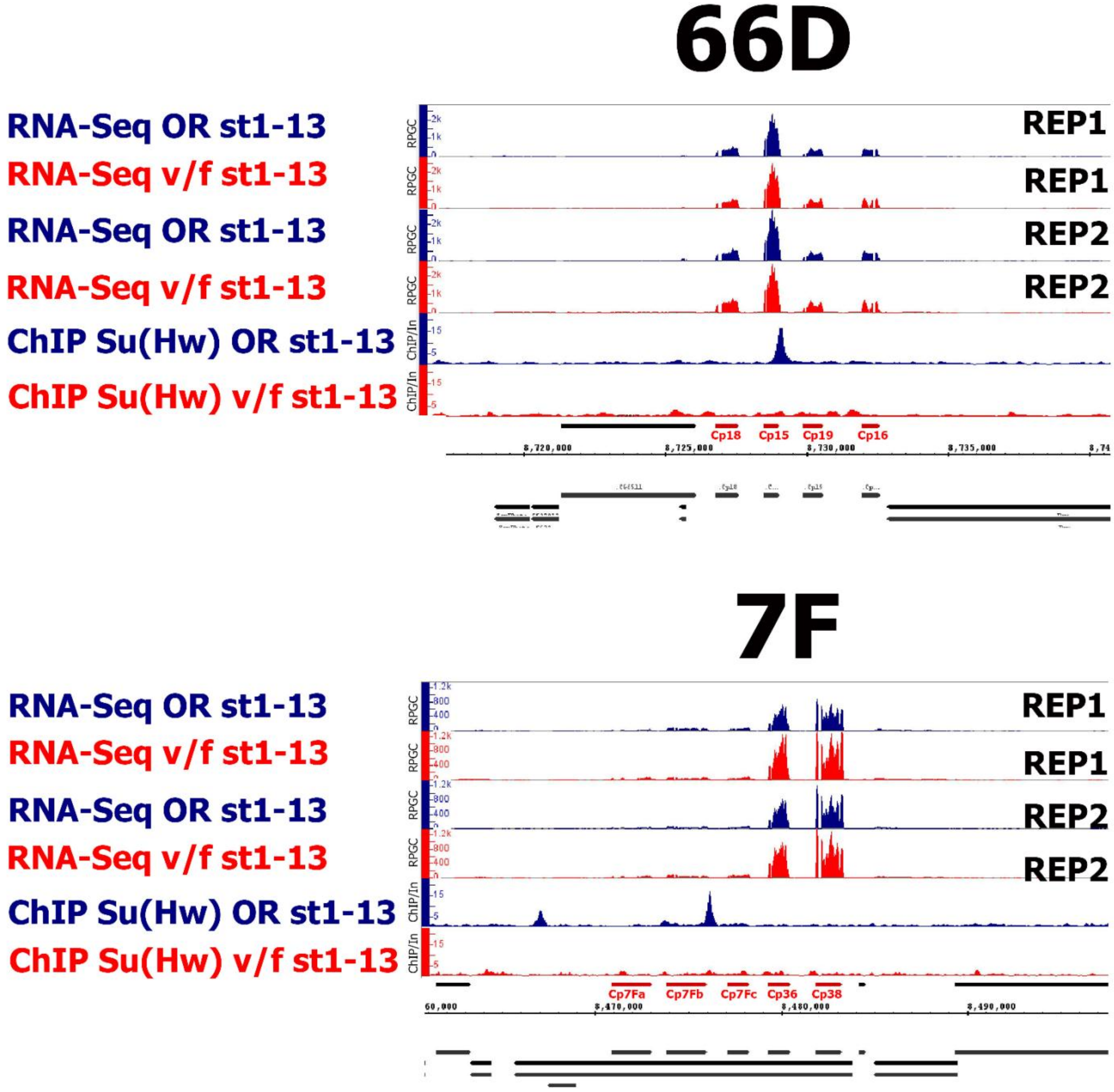

Supplementary figure 2. RNA-Seq profiles on 66D and 7F DAFCs in the wild type (or, blue profiles) and *su(Hw)*$^{v/f}$ (v/f, red profiles) ovaries. Su(Hw) ChIP-Seq profiles for the wild type (or, blue profiles) and *su(Hw)*$^{v/f}$ (v/f, red profiles) ovaries are also present. Only the ovaries containing egg chambers stages 1-13 were selected for analysis. For RNA-Seqs two replicates of each sample are present (rep1, rep2). Su(Hw) binding level was estimated by ChIP-Seq and represent the enrichment of ChIP-Seq signal over input. The coordinates of the X-axis correspond to the dm6 version of the *Drosophila* genome. The coordinates of the Y-axis represent the number of reads for RNA-Seq and the ChIP/Input ratio for ChIP-Seqs.

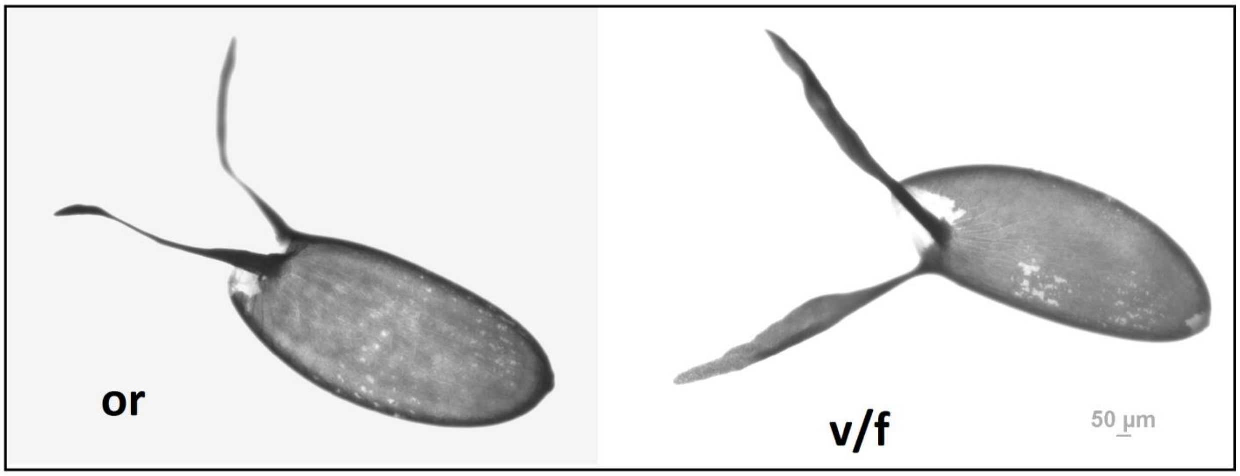

Supplementary figure 3. The phenotype of the wild-type (or) and *su(Hw)*$^{v/f}$ (v/f) embryos. The images were obtained through a light microscopy. Scale Bar: 50 µm.

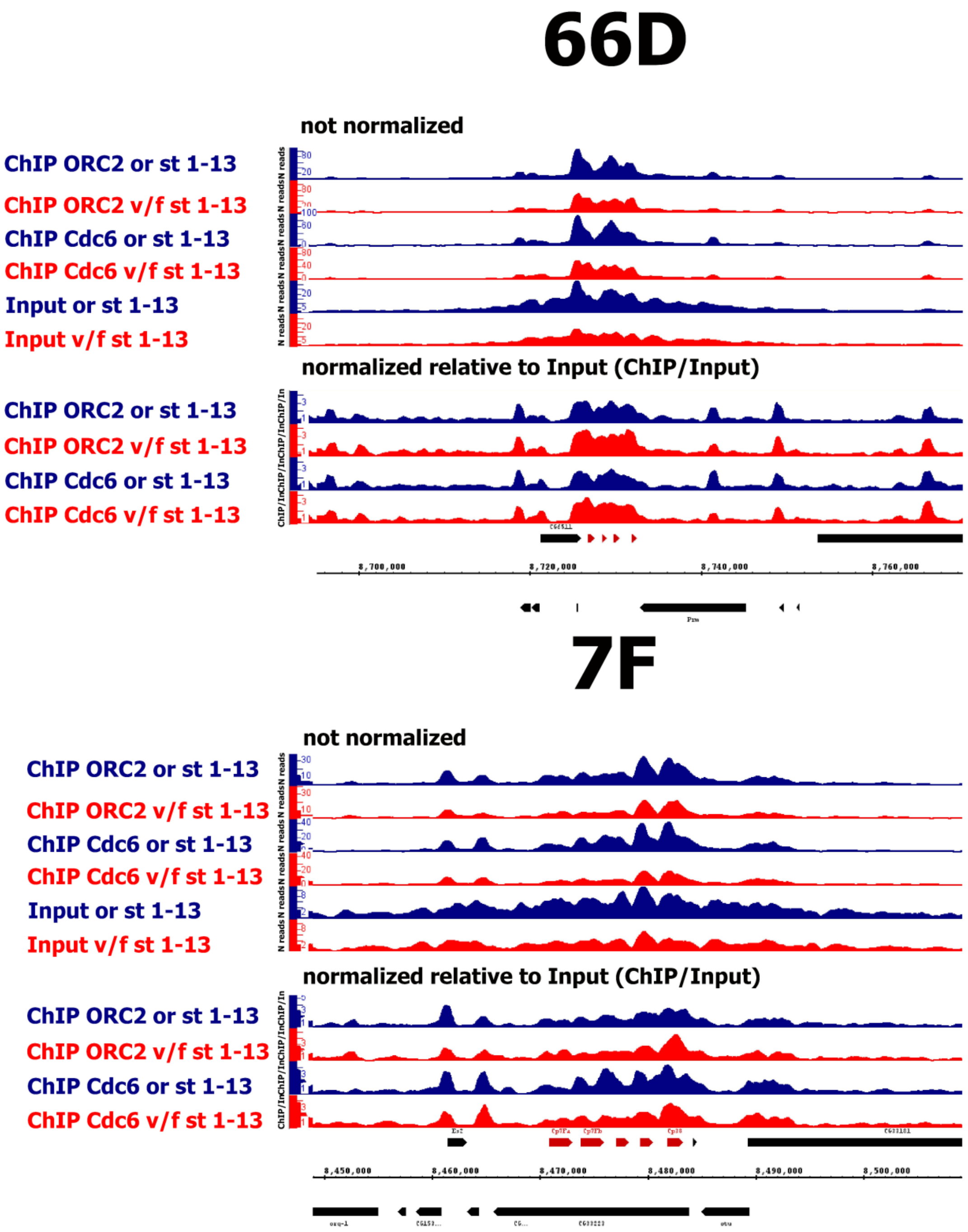

Supplementary figure 4. The binding profiles of ORC2 and CDC6 replication proteins on 66D and 7F DAFCs and on 62D insulator in the wild-type (or, blue plots) and *su(Hw)*$^{v/f}$ (v/f, red plots) ovaries. Only the ovaries containing egg chambers stages 1-13 were selected for analysis. Protein binding levels were estimated by ChIP-Seq. The ChIP-Seq and Input profiles are presented before (not normalized) and after normalization (normalized relative to Input, ChIP/Input). The coordinates of the X-axis correspond to the dm6 version of the *Drosophila* genome. The coordinates of the Y-axis represent the number of reads or the ChIP/Input ratio. The main chorionic genes of the loci are marked in red.

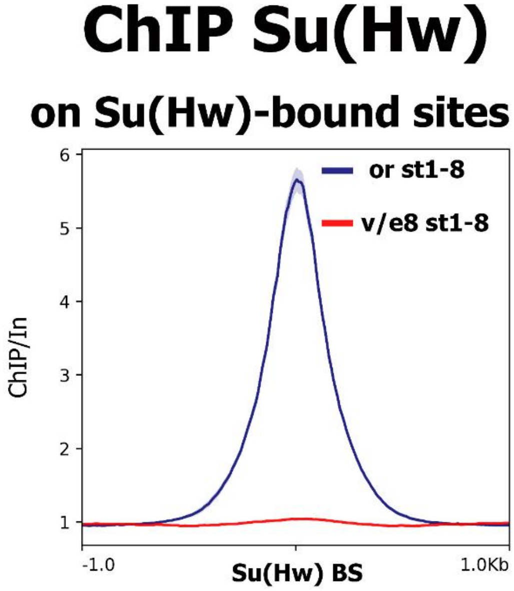

Supplementary figure 5. Average distribution of Su(Hw) binding on Su(Hw)-bound sites in the wild-type (or, blue profiles) and *su(Hw)*$^{v/E8}$ (v/e8, red profiles) ovaries. The list of Su(Hw)-bound sites was calculated basing on Su(Hw) ChIP-Seq data for the wild-type ovaries of stages 1-8. Su(Hw) binding level was calculated as an enrichment (ratio of corresponding ChIP-Seq signal over input DNA). Average profiles were calculated as a median of Su(Hw) binding levels. The standard error is displayed on the graphs as lighter area around the main line of the profiles.

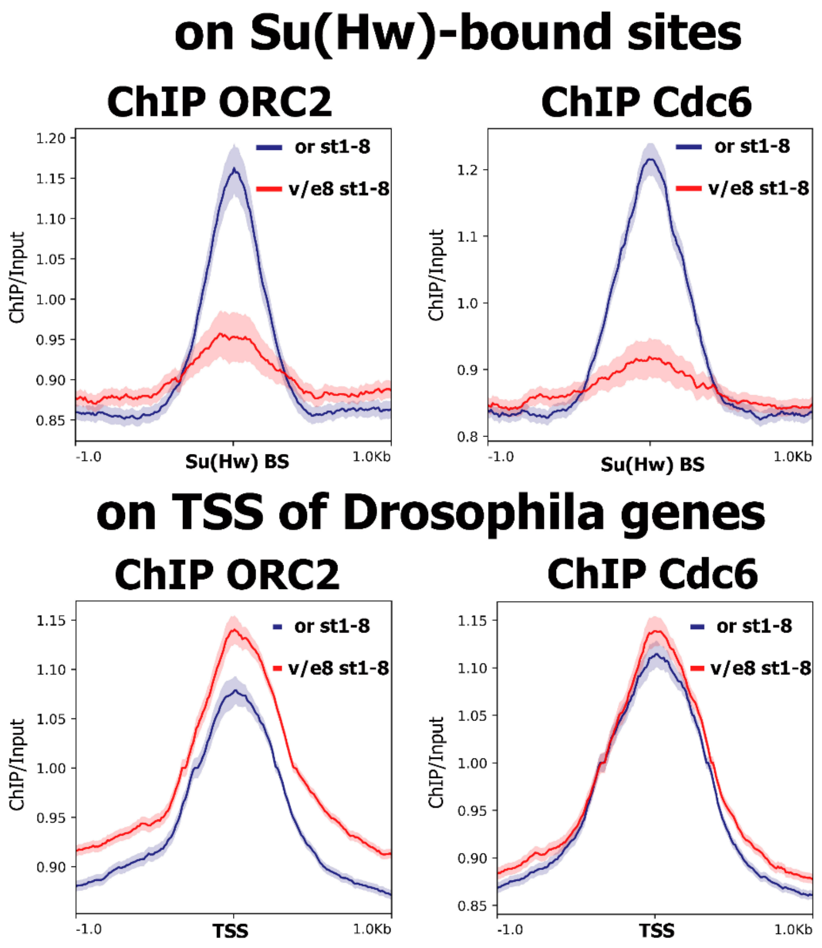

Supplementary figure 6. Average distribution of ORC2 and CDC6 replication proteins on Su(Hw)-bound sites and all TSSs in the wild-type (or, blue profiles) and *su(Hw)*$^{v/E8}$ (v/e8, red profiles) ovaries. The list of Su(Hw)-bound sites was calculated basing on our Su(Hw) ChIP-Seq data for the wild-type ovaries of stages 1-8. ORC2 and CDC6 binding levels were calculated as an enrichment (ratio of corresponding ChIP-Seq signal over input DNA). Average profiles were calculated as a median of ORC2 and CDC6 binding levels. The standard error is displayed on the graphs as lighter area around the main line of the profiles.

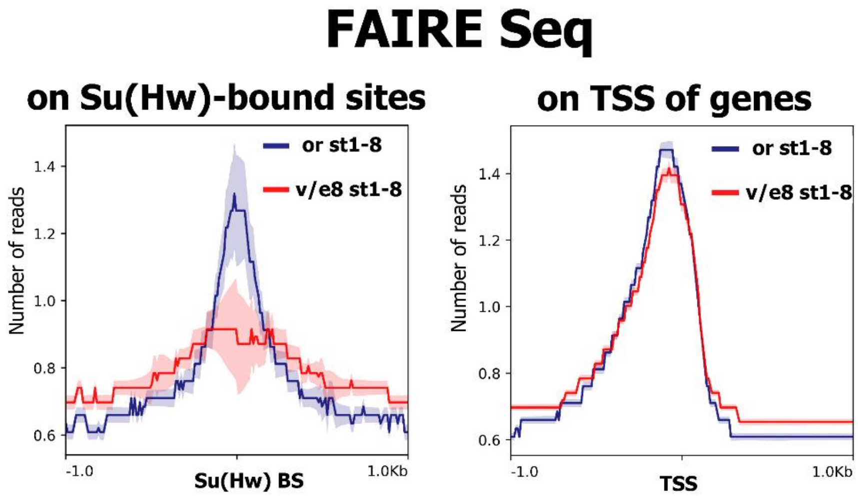

Supplementary figure 7. Average distribution of FAIRE-Seq signal on Su(Hw)-bound sites and all TSSs in the wild-type (or, blue profiles) and *su(Hw)*$^{v/E8}$ (v/e8, red profiles) ovaries. The list of Su(Hw)-bound sites was calculated basing on our Su(Hw) ChIP-Seq data for the wild-type ovaries of stages 1-8. Average profiles were calculated as a median of FAIRE-Seq signal. The standard error is displayed on the graphs as lighter area around the main line of the profiles.

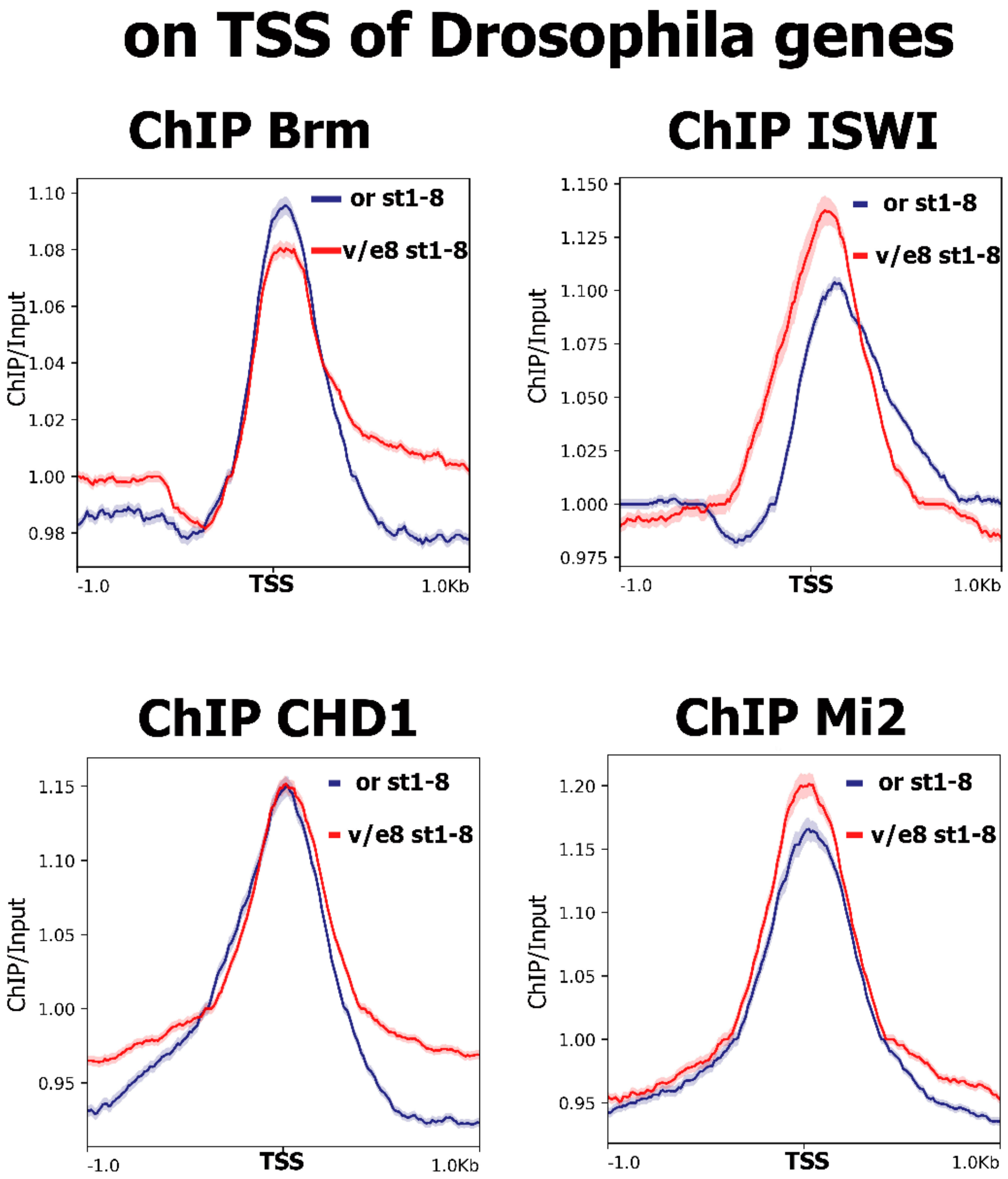

Supplementary figure 8. Average distributions of Brm, ISWI, Mi-2 and CHD1 remodelers on all TSSs in the wild-type (or, blue profiles) and *su(Hw)*$^{v/E8}$ (v/e8, red profiles) ovaries. Remodelers' binding levels were calculated as an enrichment (ratio of corresponding ChIP-Seq signal over input DNA). Average profiles were calculated as a median of Brm, ISWI, Mi-2 and CHD1 binding levels. The standard error is displayed on the graphs as lighter area around the main line of the profiles.

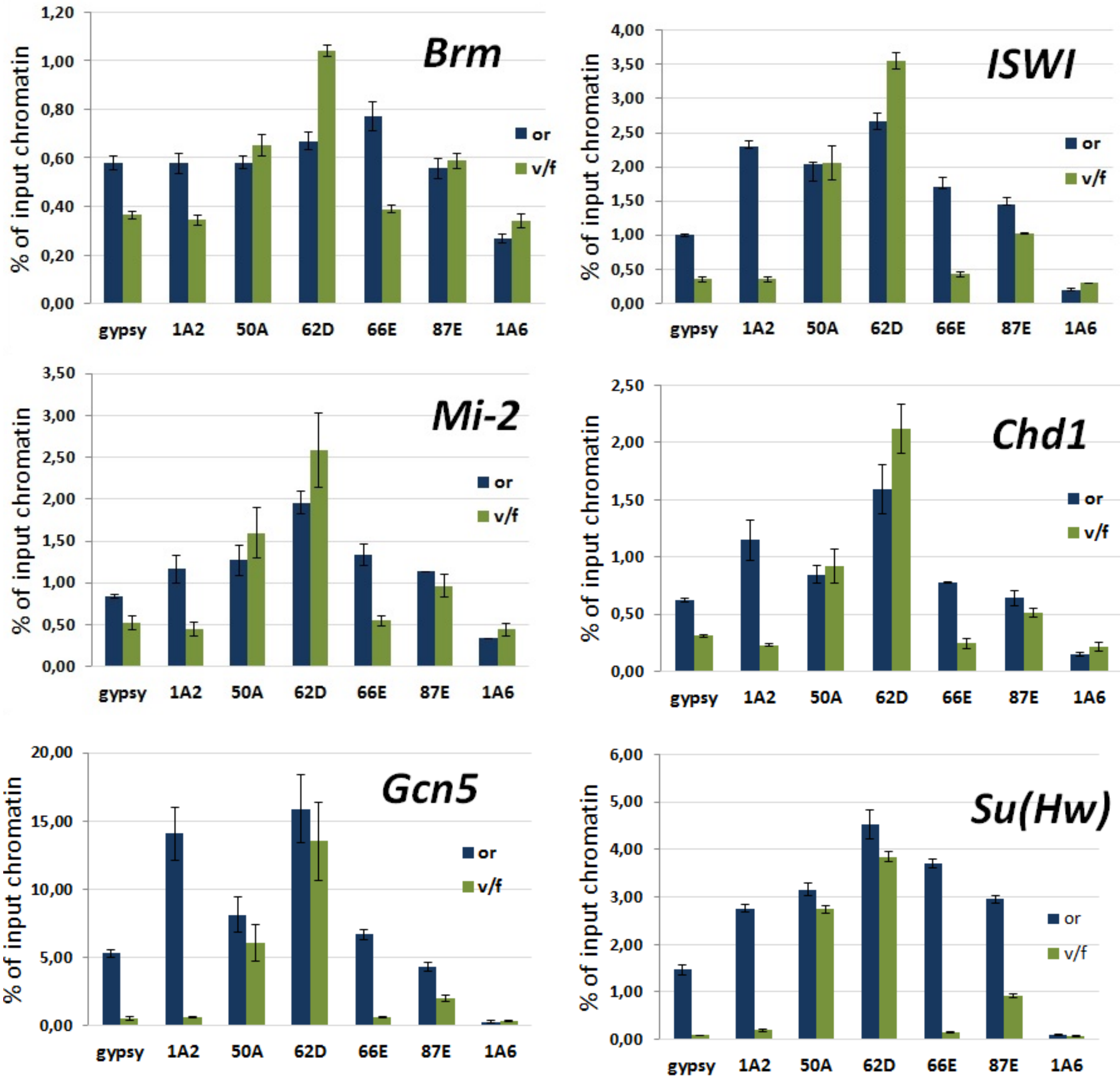

Supplementary figure 9. ChIP analysis of Brm, ISWI, Mi-2, CHD1, Gcn5 and Su(Hw) binding to some SBSs (from [9]) in the wild-type (or) and *su(Hw)*$^{v/f}$ (v/f) ovaries of 1-13 egg chamber stages assessed by qRT-PCR. The Y-axis represents the % of Input chromatin fraction. 1A6 region was used as negative control. The data are mean values from three independent experiments, error bars represent standard deviations.

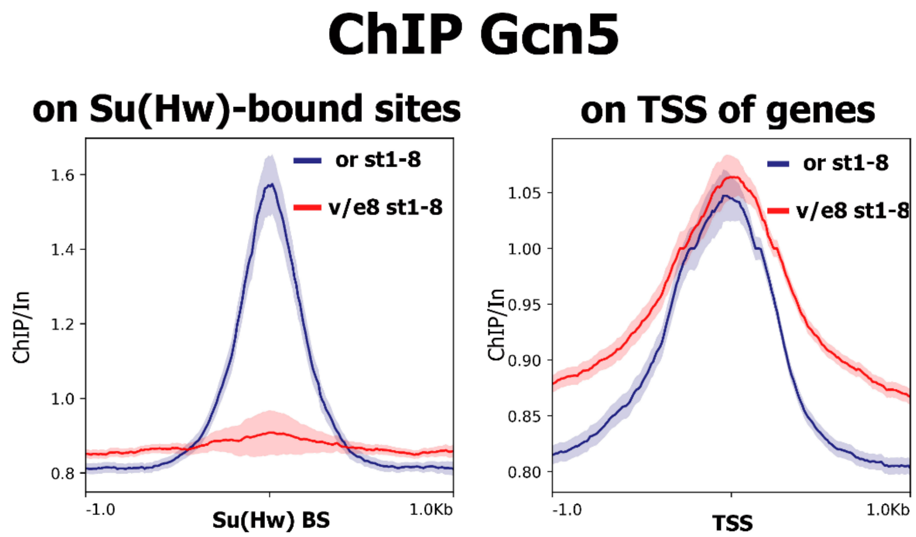

Supplementary figure 10. Average distribution of Gcn5 acetyltransferase on Su(Hw)-bound sites and all TSSs in the wild-type (or, blue profiles) and *su(Hw)*$^{v/E8}$ (v/e8, red profiles) ovaries. The list of Su(Hw)-bound sites was calculated basing on our Su(Hw) ChIP-Seq data for the wild-type ovaries of stages 1-8. Gcn5 binding levels were calculated as an enrichment (ratio of corresponding ChIP-Seq signal over input DNA). Average profiles were calculated as a median of Gcn5 binding levels. The standard error is displayed on the graphs as lighter area around the main line of the profiles.

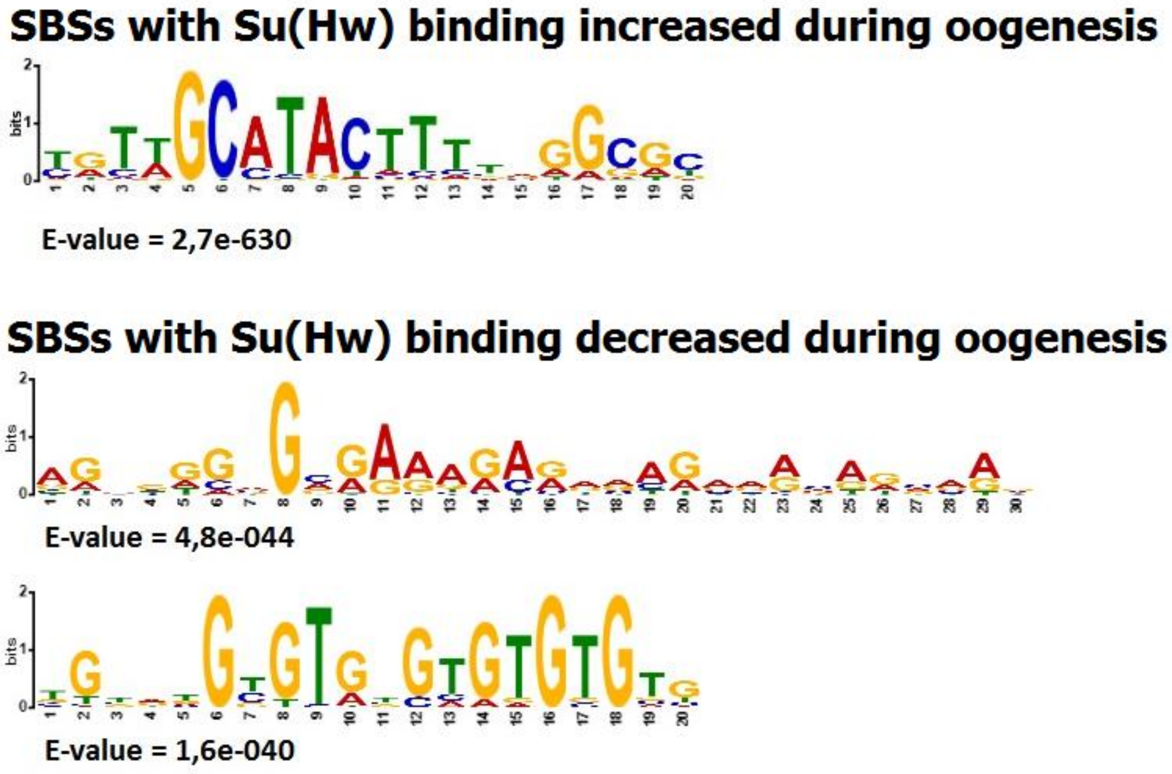

Supplementary figure 11. MEME-generated consensus sequences for SBSs with Su(Hw) binding increased and decreased during oogenesis.

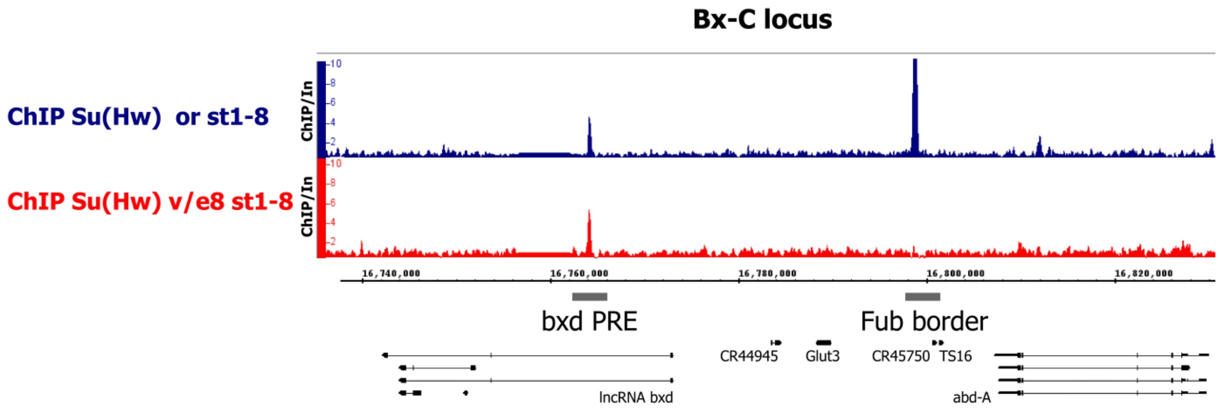

Supplementary figure 12. Su(Hw) binding profile on Bx-C locus in the wild-type (or, blue plots) and *su(Hw)*$^{v/E8}$ (v/e8, red plots) ovaries. Only the ovaries containing egg chambers stages 1-8 were selected for analysis. Protein binding levels were estimated by ChIP-Seq and represent the enrichment of ChIP-Seq signal over input. The coordinates of the X-axis correspond to the dm6 version of the *Drosophila* genome. The coordinates of the Y-axis represent the ChIP/Input ratio.

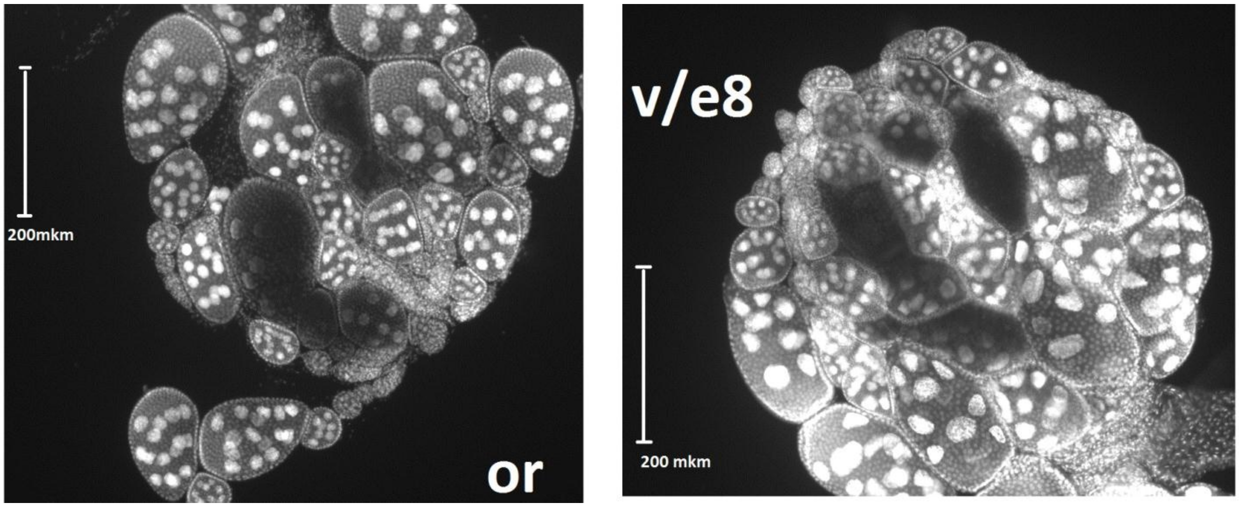

Supplementary figure 13. DAPI-stained ovaries isolated from the wild-type (or) and *su(Hw)*$^{v/E8}$ (v/e8) females 15 hours after eclosion (include egg chamber stages 1-8). Scale bars: 200 µm.

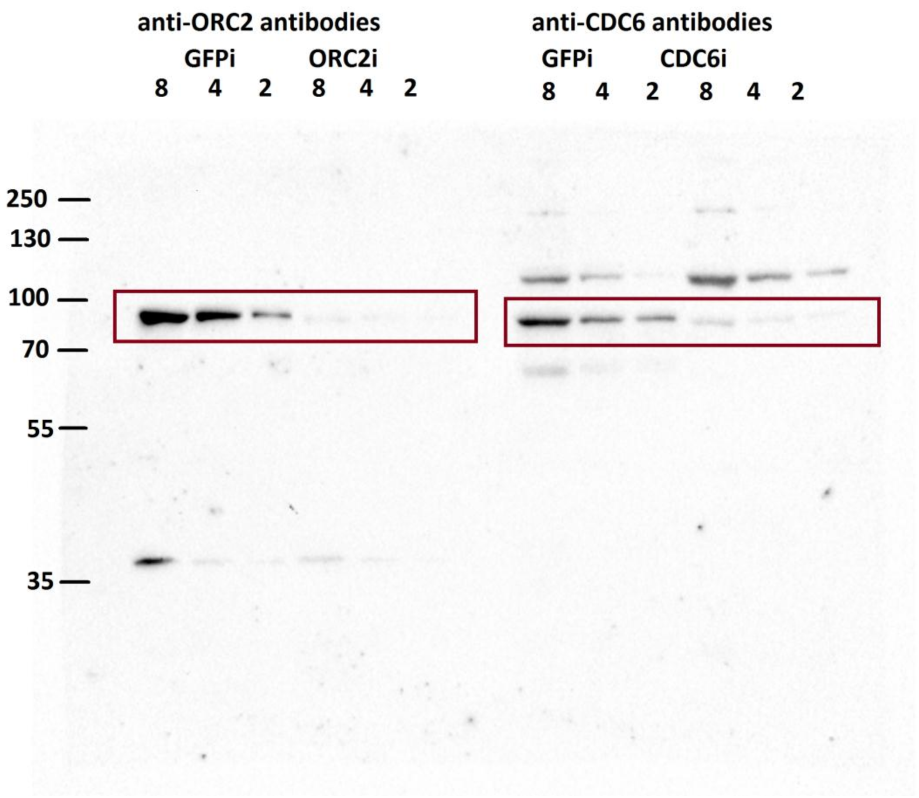
anti-ORC2 antibodies
GFPi
ORC2i
8 4 2 8 4 2
anti-CDC6 antibodies
GFPi
CDC6i
8 4 2 8 4 2
250
130
100
70
55
35

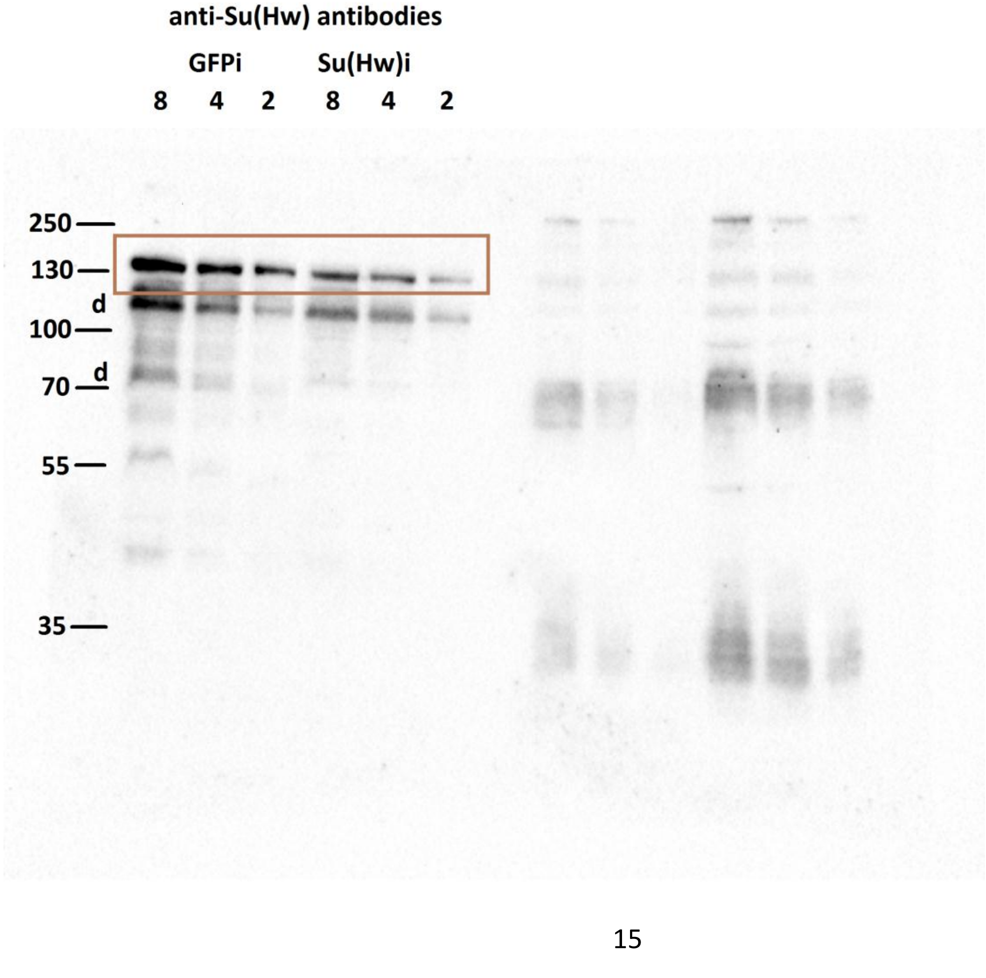
anti-Su(Hw) antibodies
GFPi
Su(Hw)i
8 4 2 8 4 2
250
130
d
100
d
70
55
35

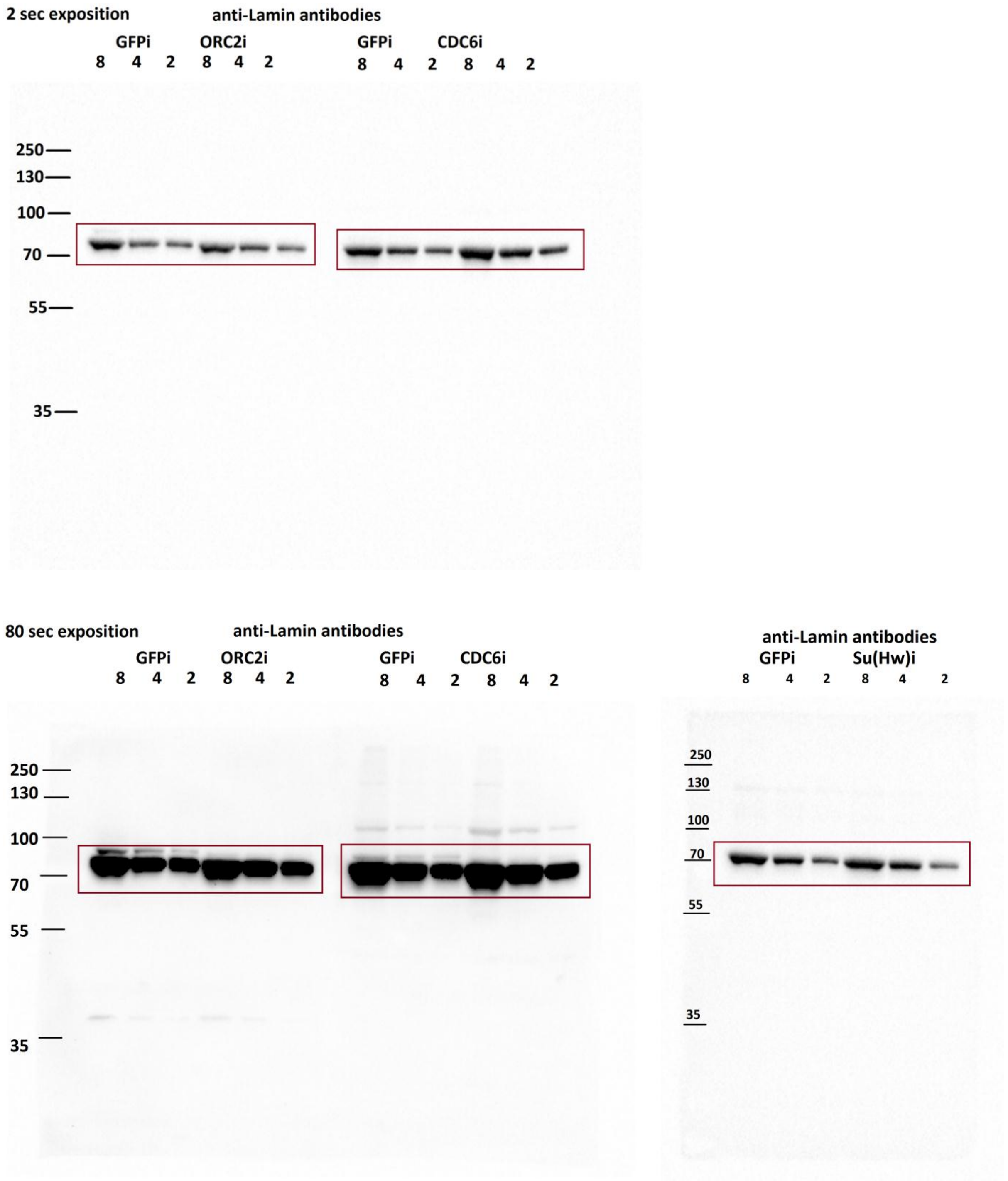

Supplementary figure 14. The test of antibodies against Su(Hw), ORC2 and CDC6. The depletion of corresponding proteins with RNA-interference was used to estimate the specificity of antibodies. Nuclear extracts from S2 *Drosophila* cells, treated with corresponding dsRNA (ORCi, CDC6i and Su(Hw)i), and dsRNA to GFP transcript (GFPi) were loaded. The antibodies, used for blot staining, are marked on the top of the figure. Numbers above the figure represent the relative portion of a loaded fraction. Anti-lamin staining was used as loading control. In Su(Hw) staining d indicate the Su(Hw) protein decay. The brown frames indicate the regions of specific antibody staining.

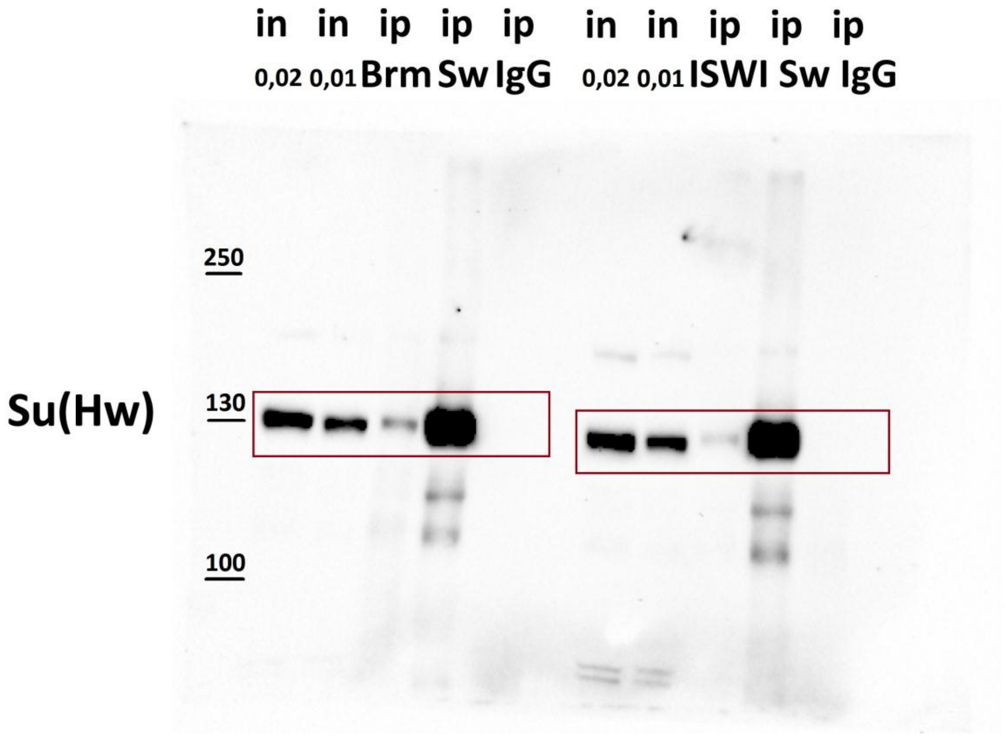
in in ip ip ip in in ip ip ip
0,02 0,01 Brm Sw IgG 0,02 0,01 ISWI Sw IgG
250
Su(Hw)
130
100

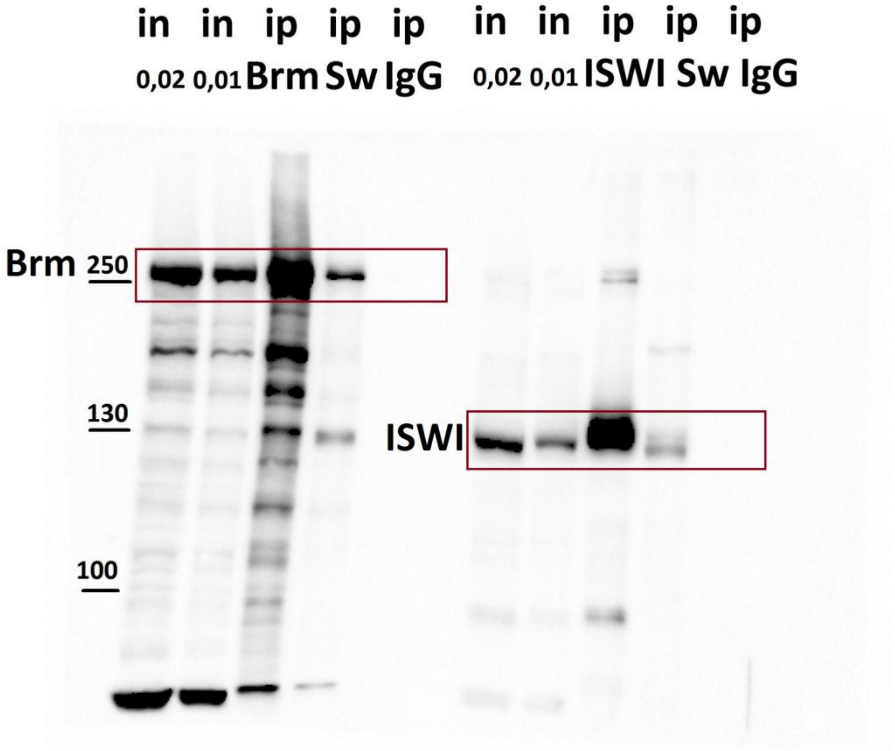
in in ip ip ip in in ip ip ip
0,02 0,01 Brm Sw IgG 0,02 0,01 ISWI Sw IgG
Brm
250
130
ISWI
100

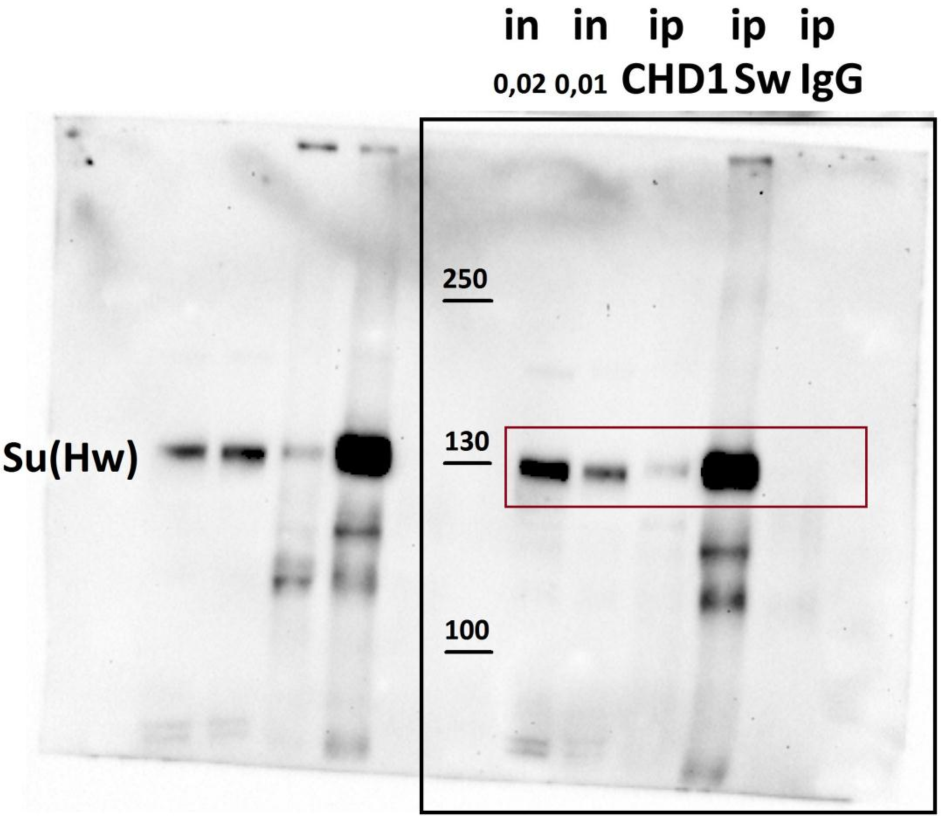
in in ip ip ip
0,02 0,01 CHD1 Sw IgG
250
Su(Hw)
130
100

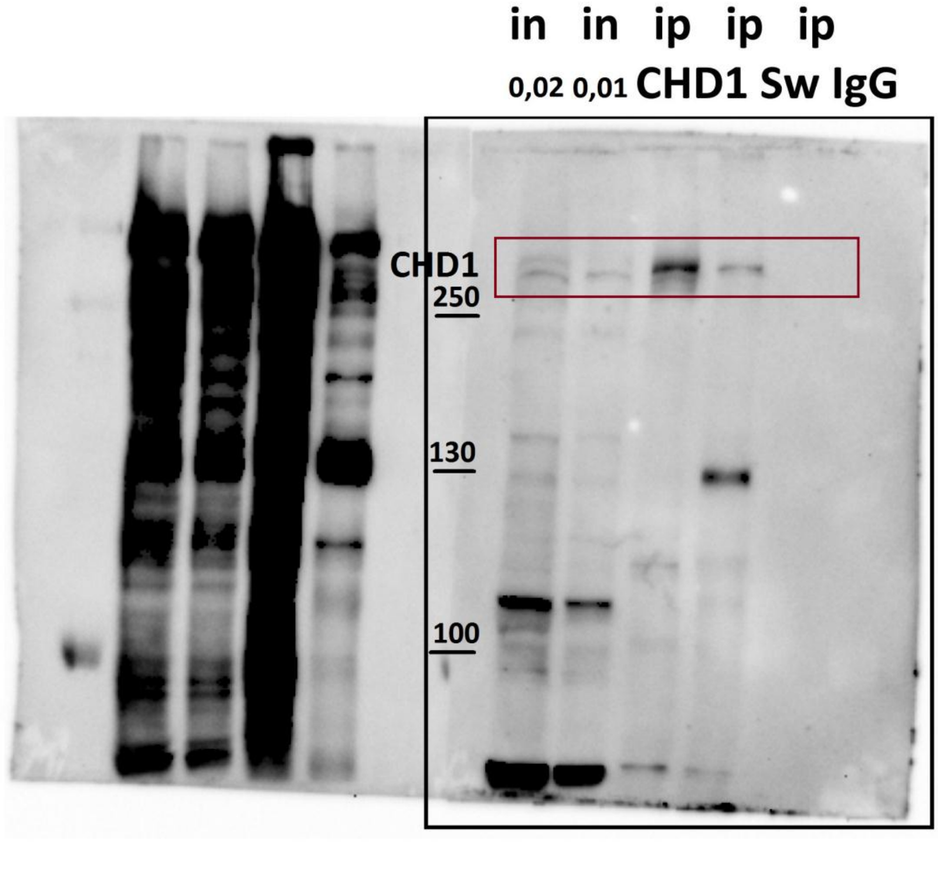
in in ip ip ip
0,02 0,01 CHD1 Sw IgG
CHD1
250
130
100

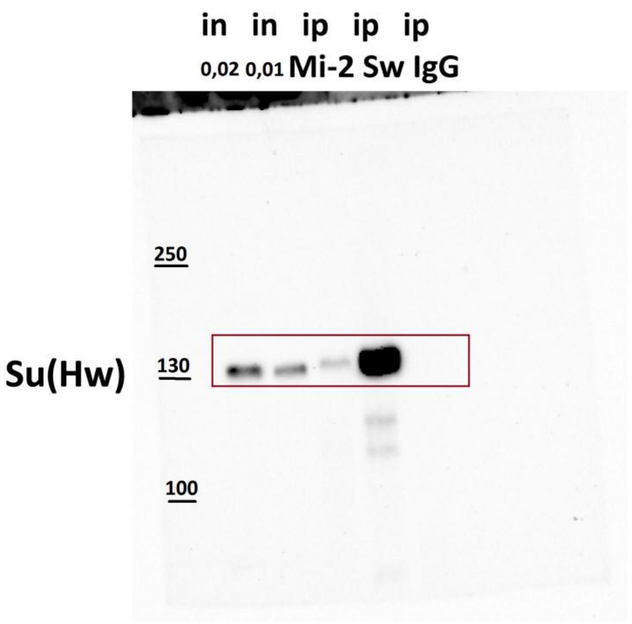
in in ip ip ip
0,02 0,01 Mi-2 Sw IgG
250
Su(Hw)
130
100

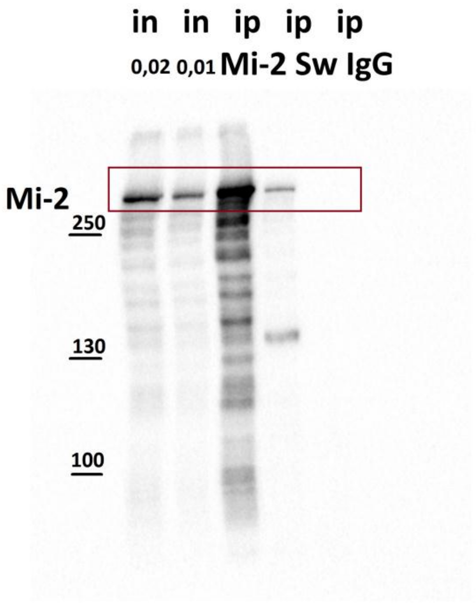
in in ip ip ip
0,02 0,01 Mi-2 Sw IgG
Mi-2
250
130
100

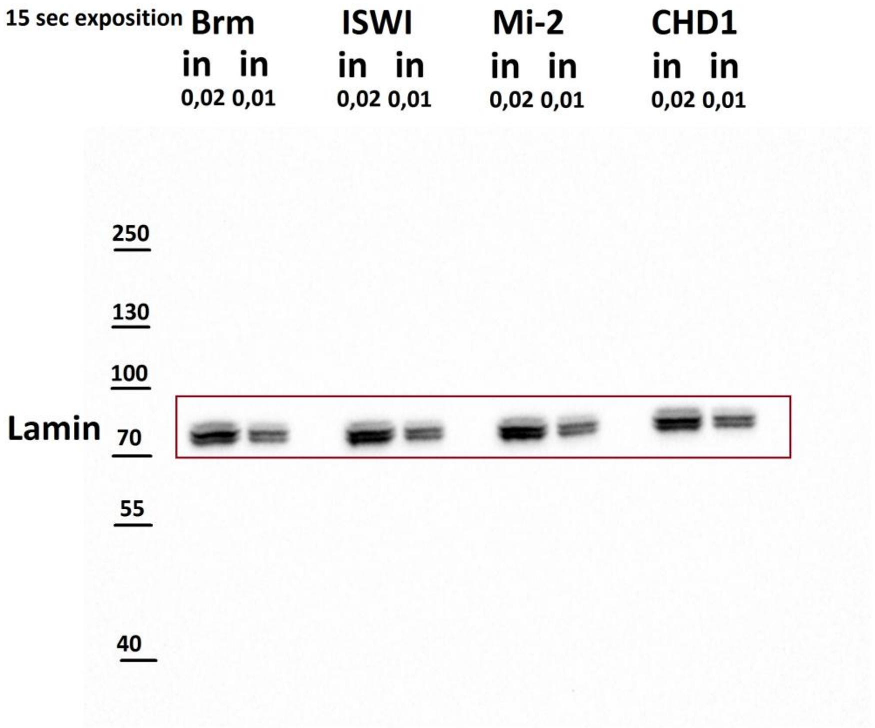
15 sec exposition
Brm ISWI Mi-2 CHD1
in in in in in in in in
0,02 0,01 0,02 0,01 0,02 0,01 0,02 0,01
250
130
100
Lamin
70
55
40

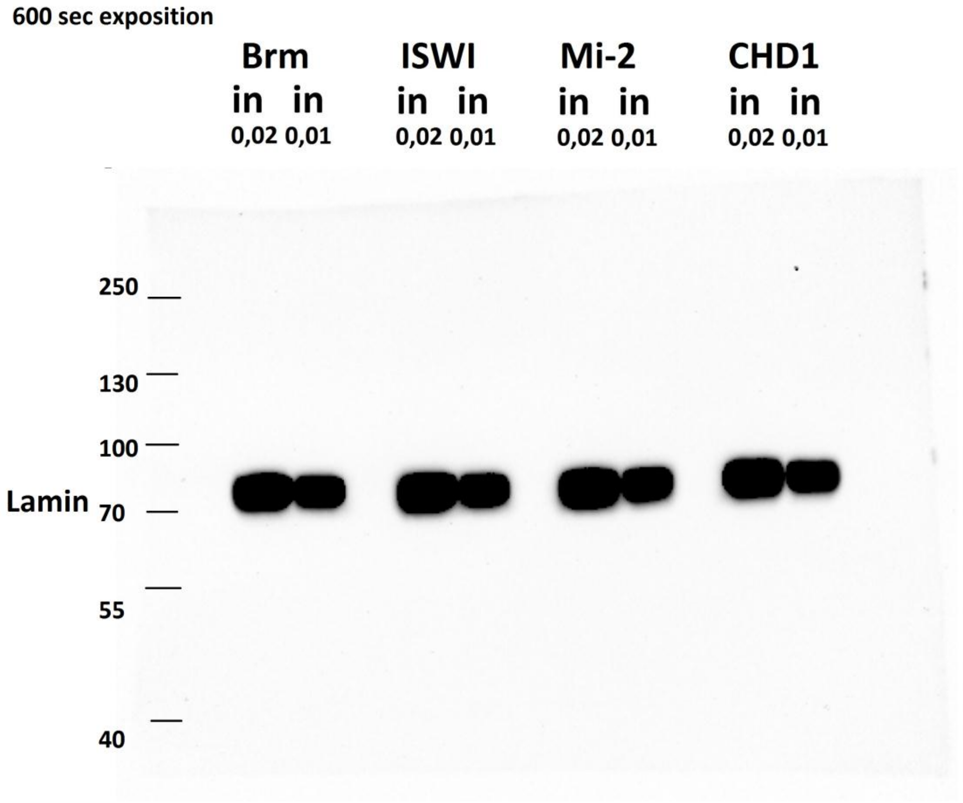

Supplementary figure 15. The original Western blots used in Fig. 3A. The IPs were performed with antibodies against Brm, ISWI, Mi-2, CHD1 remodelers and Su(Hw) (a serum of non-immunized rabbits (ip IgG) was used as a negative control), which is indicated on the top of the figure. Western blots were stained with the corresponding antibodies indicated on the left of the figures. Anti-lamin staining was used as loading control. All input and IP samples were loaded on a single western blot. The numbers above the inputs represent a portion of a loaded fraction (in respect to the amount used for immunoprecipitations). The brown frames indicate the regions of the blots with specific antibody staining that were presented at Fig. 3A.

Table S1. Differentially expressed transcripts in *su(Hw)*$^{v/f}$ (v-f) ovaries compared to the wild type (or). Only the ovaries containing egg chambers stages 1-13 were selected for RNA-Seq analysis. Two replicates of each sample were analyzed.

Gene	gene_id	or	v-f	log2(v-f/or) (fold_change)	p_value	q_value
SkpC	NM_134514	2,55933	127,699	5,64084	0,00005	0,009457
CG2993	NM_141483	0,63494	14,5645	4,51969	0,00005	0,009457
CG13366	NM_130492	1,55255	26,7216	4,10529	0,00005	0,009457
CG17224	NM_001273048	6,46416	99,6709	3,94664	0,00005	0,009457
RhoGAP100F	NM_143645	0,12344	1,79303	3,86052	0,00005	0,009457
l(2)03659	NM_001273894	0,618887	8,02305	3,6964	0,00005	0,009457
lncRNA:CR46064	NR_133104	0,543701	5,52906	3,34615	0,00005	0,009457
pyd3	NM_141475	6,51027	44,6664	2,7784	0,00005	0,009457
alpha-Man-Ia	NM_167224	1,49	10,2169	2,77758	0,00005	0,009457
CG13321	NM_136987	1,91634	9,96849	2,37902	0,00005	0,009457
Rab27	NM_130565	6,82447	33,1916	2,28203	0,00005	0,009457
CG13088	NM_135390	2,98322	13,362	2,16319	0,00005	0,009457
CG1124	NM_141252	5,37988	18,918	1,81411	0,00005	0,009457
CG31928	NM_001272940	32,9194	108,039	1,71455	0,00005	0,009457
GstD5	NM_080175	5,46112	17,5592	1,68496	0,00005	0,009457
CG10492	NM_136093	7,62022	22,0422	1,53237	0,00005	0,009457
Adk3	NM_143738	11,6892	33,7816	1,53106	0,00005	0,009457
Dip-B	NM_169557	66,861	191,375	1,51717	0,0002	0,030263
Inx7	NM_176699	5,60351	15,6268	1,47962	0,00005	0,009457
CG4250	NM_137859	30,3509	72,8329	1,26285	0,0001	0,017084
CG3842	NM_206633	12,8028	28,0665	1,13239	0,00005	0,009457
emei	NM_140182	30,7811	65,6917	1,09367	0,00005	0,009457
CG42699	NM_132035	3,91415	8,08181	1,04598	0,0002	0,030263
CG31661	NM_164420	42,1229	85,4343	1,02021	0,00005	0,009457
CG5958	NM_135275	55,1767	105,776	0,938888	0,00005	0,009457
bam	NM_057452	15,6072	29,7295	0,929683	0,00025	0,037472
CG31926	NM_164419	45,5239	86,6586	0,928721	0,00005	0,009457
Acsf2	NM_135080	17,0407	32,039	0,910844	0,0002	0,030263
MtnA	NM_079575	291,159	523,62	0,846713	0,00035	0,049211
ImpL2	NM_001274480	63,9391	34,7988	-0,87766	0,0001	0,017084
CG44774	NM_001103410	73,0008	38,3441	-0,92891	0,00005	0,009457
gammaTub37C	NM_057574	90,1559	46,3915	-0,95856	0,00015	0,024569
CG4842	NM_140628	80,553	41,2526	-0,96545	0,0001	0,017084
tko	NM_080311	200,454	101,295	-0,9847	0,00005	0,009457

DnaJ-1	NM_079212	227,153	111,371	-1,0283	0,00005	0,009457
28SrRNA:CR45844	NR_133562	22,9888	11,2225	-1,03454	0,00005	0,009457
CG30440	NM_136328	10,2501	4,88162	-1,07021	0,00035	0,049211
GILT3	NM_142908	84,0174	37,6511	-1,158	0,00015	0,024569
Pa1	NM_132484	40,3299	16,877	-1,25679	0,00005	0,009457
CG6357	NM_137063	30,7341	12,8165	-1,26183	0,00005	0,009457
CG33774	NM_001032225	353,402	147,197	-1,26356	0,0003	0,043331
CG4594	NM_135479	33,9938	14,0549	-1,2742	0,00005	0,009457
Mur18B	NM_133136	53,3299	21,7984	-1,29072	0,00005	0,009457
CG14292	NM_142502	53,5317	21,8737	-1,29119	0,00005	0,009457
dgo	NM_136770	5,08224	1,94486	-1,38579	0,0003	0,043331
CG7730	NM_176343	31,4714	11,6859	-1,42927	0,00005	0,009457
Claspin	NM_001300005	9,15659	3,3969	-1,43059	0,0002	0,030263
CG8097	NM_001298350	27,4199	9,65674	-1,50561	0,00005	0,009457
CG6678	NM_142739	12,4749	4,15521	-1,58604	0,00005	0,009457
lncRNA:Hsromega	NR_002069	116,258	34,4734	-1,75378	0,00005	0,009457
CG12112	NM_132261	57,8331	16,9901	-1,7672	0,00005	0,009457
E(spl)mbeta-HLH	NM_079781	16,5652	4,80271	-1,78623	0,00005	0,009457
CG31997	NM_166735	78,4226	22,4696	-1,80329	0,00005	0,009457
asRNA:CR44031	NR_073624	64,507	17,4499	-1,88623	0,0002	0,030263
CG31999	NM_166745	4,10944	1,10586	-1,89378	0,0002	0,030263
Hsp70Bb	NM_080188	8,90245	2,22384	-2,00115	0,00005	0,009457
Jon65Aiv	NM_139755	23,3558	5,25803	-2,15118	0,00005	0,009457
CG7408	NM_001169992	6,14217	1,37092	-2,16361	0,00035	0,049211
Hsp22	NM_001031943	263,213	57,6365	-2,19118	0,00005	0,009457
CG33946	NM_001316477	60,7129	13,2931	-2,19133	0,00015	0,024569
CG12374	NM_136975	9,22931	1,83902	-2,32729	0,0001	0,017084
lncRNA:alphagamma-element:CR32865	NR_002020	8,40593	1,44746	-2,53789	0,00005	0,009457
Bace	NM_135391	17,2797	2,96001	-2,5454	0,00005	0,009457
betaTry	NM_080165	22,1544	3,74605	-2,56415	0,00005	0,009457
Hsp23	NM_001259765	263,152	43,571	-2,59445	0,00005	0,009457
Jheh3	NM_137543	30,1758	4,92519	-2,61514	0,00005	0,009457
alphaTry	NM_057423	23,7616	3,50976	-2,75918	0,00005	0,009457
Hsp68	NM_079750	147,703	20,8235	-2,82642	0,00005	0,009457
CG11700	NM_132078	13,6435	1,88696	-2,85407	0,00005	0,009457
Hsp67Bc	NM_079270	17,3996	2,40291	-2,85619	0,00005	0,009457
CG1544	NM_143592	1,49578	0,201781	-2,89003	0,0002	0,030263
CG17977	NM_144144	8,86611	1,10666	-3,0021	0,00005	0,009457
Hsp70Bc	NM_141952	6,98904	0,846578	-3,04538	0,00005	0,009457
CG16798	NM_136186	3,19124	0,380052	-3,06985	0,0001	0,017084
Bbd	NM_137640	8,72088	0,980853	-3,15237	0,0001	0,017084

Obp56d	NM_137600	17,6153	1,90259	-3,21079	0,00005	0,009457
CG32625	NM_167381	69,0266	7,45066	-3,21171	0,00005	0,009457
thetaTry	NM_078969	5,80342	0,592554	-3,29189	0,0001	0,017084
Cyp6a17	NM_143761	6,96654	0,681496	-3,35367	0,00005	0,009457
Cyp6d2	NM_137854	2,91877	0,263954	-3,467	0,0002	0,030263
CG42565	NM_137858	56,6869	4,39253	-3,68989	0,00005	0,009457
PIG-A	NM_001299618	15,8107	1,19963	-3,72024	0,0003	0,043331
CG30377	NM_001299242	23,086	1,71914	-3,74726	0,00005	0,009457
alpha-Est10	NM_001260034	30,2331	2,18001	-3,79372	0,00005	0,009457
CG18628	NM_140144	80,9102	5,70914	-3,82498	0,00005	0,009457
IM33	NM_134956	35,162	2,44194	-3,84792	0,00005	0,009457
CG17108	NM_135595	6,65691	0,423839	-3,97326	0,0003	0,043331
CG2187	NM_001202402	17,0457	1,00041	-4,09075	0,00005	0,009457
Fatp3	NM_166666	38,4363	2,15365	-4,15761	0,00005	0,009457
CG2233	NM_132205	16,5063	0,748365	-4,46313	0,00005	0,009457
Hsc70-2	NM_079615	11,8608	0,402011	-4,88282	0,00005	0,009457
lncRNA:dntRL	NR_133103	20,3146	0,605446	-5,06837	0,00005	0,009457
GstZ1	NM_141637	31,9926	0,900276	-5,15123	0,00005	0,009457

Table S2. The list of SBSs with Su(Hw) binding changing during the wild-type ovary development (during the transition from the mid-oogenesis (1-8 egg chamber stages) to late-oogenesis (1-13 egg chamber stages)). Given positions correspond to dm6 version of *Drosophila melanogaster* genome.

SBSs with Su(Hw) binding:

increased during oogenesis more than two-fold				decreased during oogenesis more than two-fold			
**№**	**Chromosome**	**Position**	**Cluster**	**№**	**Chromosome**	**Position**	**Cluster**
1	3L	8729096	cluster 2	1	X	2304145	cluster 3
2	2L	7063367	cluster 2	2	X	13563635	cluster 3
3	2R	13614232	cluster 2	3	X	14978972	cluster 2
4	3R	7266670	cluster 2	4	3L	19295095	cluster 3
5	3L	8714454	cluster 2	5	2L	5403770	cluster 3
6	3R	23965086	cluster 2	6	3R	18413001	cluster 3
7	X	7384265	cluster 2	7	X	18219727	cluster 2
8	X	18050984	cluster 2	8	3L	11036216	cluster 2
9	3R	12345057	cluster 2	9	3L	15276385	cluster 2
10	X	8912038	cluster 2	10	2L	11517326	cluster 3
11	2L	2782985	cluster 2	11	X	11580711	cluster 3
12	2R	9512081	cluster 2	12	2R	21502053	cluster 3
13	2R	15004643	cluster 2	13	X	2137024	cluster 3
14	X	11769596	cluster 2	14	3R	14655032	cluster 3
15	X	23281359	cluster 2	15	3L	20621845	cluster 2
16	X	19074831	cluster 2	16	2R	18157045	cluster 3
17	X	2838803	cluster 2	17	2L	12974195	cluster 3
18	2L	16197687	cluster 2	18	2R	18760716	cluster 2
19	X	19184745	cluster 2	19	2L	13990915	cluster 2
20	2R	23912065	cluster 2	20	X	15024465	cluster 3
21	3L	7162148	cluster 2	21	3L	10898480	cluster 3
22	X	10193987	cluster 2	22	X	13278660	cluster 3
23	2R	10372129	cluster 2	23	2L	6644572	cluster 3
24	X	15969819	cluster 2	24	X	12755094	cluster 3
25	X	19415419	cluster 2	25	3R	21605583	cluster 3
26	X	9523763	cluster 2	26	X	19638422	cluster 3
27	3R	14037586	cluster 2	27	X	7938416	cluster 3
28	3L	7425670	cluster 2	28	X	3680208	cluster 3
29	3L	6577517	cluster 2	29	X	796879	cluster 3
30	3L	12331359	cluster 2	30	2L	3144849	cluster 3
31	2L	20255095	cluster 2	31	3R	18573099	cluster 3
32	X	5587967	cluster 2	32	X	8721705	cluster 3
33	X	10314022	cluster 2	33	2L	20823862	cluster 3
34	2L	10154584	cluster 2	34	2L	7418413	cluster 3
35	X	23537889	cluster 2	35	2R	6873073	cluster 3

36	2L	5865843	cluster 2	36	X	8421364	cluster 3
37	X	7390168	cluster 2	37	3R	12998131	cluster 3
38	X	14414635	cluster 2	38	3L	1489029	cluster 2
39	2L	3719663	cluster 2	39	2L	14689174	cluster 3
40	X	12966340	cluster 2	40	3R	4821436	cluster 3
41	3L	1148952	cluster 2	41	2R	13942266	cluster 3
42	X	8467082	cluster 2	42	4	490331	cluster 2
43	3L	23219916	cluster 2	43	2R	11593439	cluster 3
44	X	19033949	cluster 2	44	2L	12546764	cluster 3
45	3R	28668928	cluster 2	45	3R	16869495	cluster 3
46	X	19094043	cluster 2	46	X	686794	cluster 3
47	2L	10759959	cluster 2	47	2R	18777540	cluster 3
48	3L	9169090	cluster 2	48	X	6803472	cluster 3
49	X	15254416	cluster 2	49	X	11292269	cluster 2
50	3L	10193330	cluster 2	50	3R	13196123	cluster 2
51	2L	6570063	cluster 2	51	2L	10506889	cluster 3
52	2R	7986135	cluster 2	52	X	6352190	cluster 3
53	X	7748551	cluster 2	53	X	4909138	cluster 3
54	X	19040440	cluster 2	54	3R	10882232	cluster 3
55	3R	17506765	cluster 2	55	X	2294563	cluster 3
56	X	18144047	cluster 2	56	3L	2636156	cluster 3
57	2L	4318177	cluster 2	57	3R	17534640	cluster 3
58	2L	12958074	cluster 2	58	3R	21619318	cluster 3
59	2R	22617541	cluster 2	59	X	8061122	cluster 3
60	X	18261729	cluster 2	60	2L	14490713	cluster 3
61	2R	12830235	cluster 2	61	3L	20518690	cluster 3
62	2R	20840799	cluster 2	62	3L	14427379	cluster 3
63	X	13391283	cluster 2	63	3R	566987	cluster 2
64	X	12076762	cluster 2	64	3R	18021457	cluster 3
65	2R	22945145	cluster 2	65	2R	15728639	cluster 2
66	3R	26707371	cluster 2	66	3R	14026334	cluster 2
67	2L	20267265	cluster 2	67	3R	13398413	cluster 2
68	2L	13632041	cluster 2	68	3R	12627865	cluster 3
69	3R	19187800	cluster 2	69	3R	5648374	cluster 3
70	2R	10273799	cluster 2	70	2L	13842323	cluster 1
71	3R	30520587	cluster 2	71	2L	15062174	cluster 3
72	3L	16019336	cluster 2	72	2R	7245817	cluster 3
73	X	13992882	cluster 2	73	2R	12996377	cluster 3
74	X	15402100	cluster 2	74	3R	6893247	cluster 2
75	X	9813454	cluster 2	75	X	5560782	cluster 3
76	2L	5932069	cluster 2	76	X	2776618	cluster 2

77	3L	13688632	cluster 2	77	2L	15189645	cluster 2
78	2R	21234292	cluster 2	78	3R	18436968	cluster 2
79	X	19899395	cluster 2	79	X	3371437	cluster 3
80	X	19037318	cluster 2	80	2R	12588286	cluster 3
81	2L	13437104	cluster 2	81	2R	11193870	cluster 2
82	X	20502993	cluster 2	82	X	4026588	cluster 2
83	2R	8301781	cluster 2	83	X	629424	cluster 3
84	2L	10475766	cluster 2	84	3L	2635706	cluster 3
85	2R	21457237	cluster 2	85	3L	1517743	cluster 3
86	2L	8973117	cluster 2	86	3R	4378889	cluster 3
87	3R	6254754	cluster 2	87	X	12760549	cluster 3
88	2R	13367361	cluster 2	88	2L	4019963	cluster 1
89	3L	5919155	cluster 2	89	3R	31608909	cluster 3
90	X	850160	cluster 2	90	3R	9236157	cluster 3
91	X	13207269	cluster 2	91	3L	15589011	cluster 3
92	X	4858127	cluster 2	92	2R	12529456	cluster 3
93	X	10280134	cluster 2	93	2L	12272412	cluster 2
94	2R	7008727	cluster 2	94	2R	15573851	cluster 3
95	3R	17932849	cluster 2	95	2L	13932001	cluster 2
96	2R	23504321	cluster 2	96	2R	13015821	cluster 3
97	2L	3983491	cluster 2	97	3L	16884314	cluster 2
98	3L	10013664	cluster 2	98	2L	18152499	cluster 3
99	3L	17349875	cluster 2	99	X	5085690	cluster 3
100	3R	19972298	cluster 2	100	X	13384009	cluster 3
101	2L	14797584	cluster 2	101	X	17832342	cluster 3
102	2R	18468685	cluster 2	102	X	4910670	cluster 3
103	X	21044484	cluster 3	103	2L	19795691	cluster 2
104	2R	14954666	cluster 2	104	2R	20998109	cluster 3
105	3L	6668057	cluster 2	105	3L	224746	cluster 3
106	3R	11389915	cluster 2	106	X	6050679	cluster 3
107	X	7690697	cluster 2	107	2L	4923427	cluster 2
108	3L	22636023	cluster 2	108	3L	851258	cluster 2
109	X	12633319	cluster 2	109	2L	16287919	cluster 3
110	2R	23489007	cluster 2	110	2R	9308650	cluster 3
111	2L	10624691	cluster 2	111	X	7307744	cluster 3
112	2R	16916806	cluster 2	112	2R	6151324	cluster 3
113	2R	24439962	cluster 2	113	2L	5907440	cluster 3
114	2L	2539850	cluster 2	114	X	8193108	cluster 3
115	X	18818801	cluster 2	115	X	6968615	cluster 3
116	3L	18255701	cluster 2	116	3R	24651209	cluster 3
117	3R	8478608	cluster 2	117	3L	18019793	cluster 3

118	X	9118083	cluster 2	118	X	21042763	cluster 2
119	2L	4089915	cluster 2	119	X	11454303	cluster 3
120	X	2258866	cluster 2	120	2R	20884594	cluster 3
121	2L	15613737	cluster 2	121	X	12660116	cluster 3
122	3L	20032728	cluster 2	122	3R	27968212	cluster 1
123	X	8476154	cluster 2	123	2R	10428329	cluster 3
124	3R	25566066	cluster 2	124	2R	17145838	cluster 3
125	3L	22172295	cluster 2	125	3R	22750753	cluster 3
126	2L	5732512	cluster 2	126	X	2738773	cluster 3
127	3R	20142871	cluster 2	127	X	17886325	cluster 3
128	2L	2075522	cluster 2	128	2R	17458709	cluster 3
129	3L	12309926	cluster 2	129	3L	3250527	cluster 3
130	3R	23532943	cluster 2	130	2R	6652920	cluster 3
131	X	7154231	cluster 2	131	3R	20309534	cluster 3
132	2R	20967463	cluster 2	132	2R	11528347	cluster 2
133	X	23278545	cluster 2	133	2L	8403476	cluster 3
134	2R	15639433	cluster 2	134	3R	5088126	cluster 3
135	2L	16229737	cluster 2	135	X	13995179	cluster 3
136	X	8349137	cluster 2	136	2L	6647595	cluster 3
137	2L	16117632	cluster 2	137	3R	20283836	cluster 2
138	2L	15020188	cluster 2	138	X	1617807	cluster 3
139	2R	21235848	cluster 2	139	X	2222570	cluster 3
140	3R	21154381	cluster 2	140	2R	11639553	cluster 3
141	3R	11543464	cluster 2	141	3L	11586857	cluster 3
142	3L	2858697	cluster 2	142	3L	9695044	cluster 2
143	X	19738136	cluster 2	143	X	2732203	cluster 3
144	3L	13346452	cluster 2	144	2L	7423864	cluster 3
145	3L	9332551	cluster 2	145	3L	20771198	cluster 3
146	X	19796965	cluster 2	146	X	3162833	cluster 3
147	3R	18554826	cluster 2	147	X	13548591	cluster 2
148	2L	15833644	cluster 2	148	3R	17704106	cluster 2
149	3L	2821526	cluster 2	149	2R	7407824	cluster 3
150	2R	10720070	cluster 2	150	X	11493070	cluster 3
151	3L	11596565	cluster 2	151	X	3719809	cluster 3
152	3R	7296805	cluster 2	152	2L	16286143	cluster 3
153	X	19827613	cluster 2	153	X	19167214	cluster 2
154	3R	23017985	cluster 2	154	X	19506044	cluster 3
155	3R	17403935	cluster 2	155	3L	22266668	cluster 3
156	X	20289504	cluster 2	156	3L	20401511	cluster 3
157	2L	15778204	cluster 2	157	2L	2987775	cluster 3
158	3L	9787696	cluster 2	158	2R	9546587	cluster 3

159	3R	32015028	cluster 2	159	2R	14482348	cluster 3
160	3R	24734929	cluster 2	160	2R	14007026	cluster 3
161	3L	16181705	cluster 2	161	2L	12010942	cluster 3
162	X	19315483	cluster 2	162	3R	28102030	cluster 2
163	3L	8710876	cluster 2	163	3R	23214963	cluster 3
164	3R	6239168	cluster 2	164	3L	11118825	cluster 3
165	2L	11785236	cluster 2	165	X	7291378	cluster 3
166	3L	10080600	cluster 2	166	3L	19815884	cluster 3
167	X	16757886	cluster 2	167	2R	13223595	cluster 3
168	2R	13369085	cluster 2	168	3L	12488502	cluster 2
169	3L	6156079	cluster 2	169	3L	25574399	cluster 2
170	3R	19533474	cluster 2	170	2L	19158565	cluster 3
171	2L	261696	cluster 2	171	2R	13038968	cluster 3
172	3R	30521228	cluster 2	172	X	8625024	cluster 3
173	X	9525005	cluster 2	173	X	12919344	cluster 3
174	3R	13119034	cluster 2	174	X	12930815	cluster 3
175	2R	22989690	cluster 2	175	X	12942286	cluster 2
176	3R	20940239	cluster 2	176	3L	12953757	cluster 3
177	X	6719545	cluster 2	177	2L	12965228	cluster 3
178	3R	11833740	cluster 2	178	3R	12976699	cluster 3
179	2R	12096520	cluster 2	179	X	12988170	cluster 2
180	3R	23981257	cluster 2	180	3L	12999641	cluster 2
181	3L	16293218	cluster 2	181	3L	13011111	cluster 2
182	3R	25933969	cluster 2	182	2L	13022582	cluster 3
183	3R	12608522	cluster 2	183	X	13034053	cluster 3
184	3R	8621746	cluster 2	184	2R	13045524	cluster 3
185	3R	28496999	cluster 2	185	X	13056995	cluster 3
186	3R	14832496	cluster 2	186	3R	13068466	cluster 3
187	2L	18934979	cluster 2	187	3L	13079937	cluster 2
188	X	8979616	cluster 2	188	2R	13091408	cluster 3
189	3L	10170270	cluster 2	189	2L	13102878	cluster 3
190	2L	20248420	cluster 2	190	2R	13114349	cluster 2
191	3R	27643247	cluster 2	191	2L	13125820	cluster 2
192	3L	21186782	cluster 2	192	X	13137291	cluster 3
193	3R	14909424	cluster 2	193	3L	13148762	cluster 3
194	3L	6658551	cluster 2	194	X	13160233	cluster 3
195	2R	20449679	cluster 2	195	2L	13171704	cluster 3
196	2L	13420690	cluster 2	196	X	13183175	cluster 3
197	2L	11869115	cluster 2	197	3R	13194645	cluster 3
198	X	19162474	cluster 2	198	X	13206116	cluster 3
199	3R	9971208	cluster 2	199	X	13217587	cluster 3

200	2L	15636940	cluster 2
201	2R	13829881	cluster 2
202	3R	17454498	cluster 2
203	2R	17302769	cluster 2
204	2L	9871755	cluster 2
205	X	17521277	cluster 2
206	3R	23244478	cluster 2
207	2L	21105435	cluster 2
208	X	11917529	cluster 2
209	2R	8713048	cluster 2
210	3R	20897808	cluster 2
211	3L	8267944	cluster 2
212	3L	10481390	cluster 2
213	X	21493430	cluster 2
214	3R	29327140	cluster 2
215	2R	18160538	cluster 3
216	X	18336132	cluster 2
217	3L	13332741	cluster 2
218	X	3308229	cluster 2
219	2L	4244120	cluster 2
220	X	13483941	cluster 2
221	2R	23410365	cluster 2
222	X	6509820	cluster 2
223	X	23535634	cluster 2
224	2R	20820385	cluster 2
225	X	4778599	cluster 2
226	3R	9328993	cluster 2
227	X	13067606	cluster 2
228	3L	13644442	cluster 2
229	3R	11090080	cluster 2
230	2L	14100652	cluster 2
231	3R	23539419	cluster 2
232	3L	5664325	cluster 2
233	2R	12860625	cluster 2
234	3L	12226969	cluster 2
235	3L	11582369	cluster 2
236	2L	19849376	cluster 2
237	X	15355827	cluster 2
238	3R	31268881	cluster 2
239	2R	8270484	cluster 2
240	X	4334630	cluster 2

200	X	13229058	cluster 3
201	X	13240529	cluster 3
202	2L	13252000	cluster 3
203	3R	13263471	cluster 3
204	X	13274942	cluster 3
205	2L	13286412	cluster 3
206	2L	13297883	cluster 3
207	2R	13309354	cluster 3
208	X	13320825	cluster 3
209	3R	13332296	cluster 3
210	3L	13343767	cluster 2
211	2L	13355238	cluster 3

241	X	6180059	cluster 2
242	X	18353635	cluster 2
